# Recent Advanced Synthesis Strategies for the Nanomaterial-Modified Proton Exchange Membrane in Fuel Cells

**DOI:** 10.3390/membranes13060590

**Published:** 2023-06-09

**Authors:** Somasundaram Chandra Kishore, Suguna Perumal, Raji Atchudan, Muthulakshmi Alagan, Mohammad Ahmad Wadaan, Almohannad Baabbad, Devaraj Manoj

**Affiliations:** 1 Department of Biomedical Engineering, Saveetha School of Engineering, Saveetha Institute of Medical and Technical Sciences, Saveetha Nagar, Chennai 602105, Tamil Nadu, India; 2 Department of Chemistry, Sejong University, Seoul 143747, Republic of Korea; suguna.perumal@gmail.com; 3 School of Chemical Engineering, Yeungnam University, Gyeongsan 38541, Republic of Korea; 4 Center for Environmental Management Laboratory, National Institute of Technical Teachers Training and Research, Chennai 600113, Tamil Nadu, India; almuthulakshmi@gmail.com; 5 Department of Zoology, College of Science, King Saud University, P.O. Box 2455, Riyadh 11451, Saudi Arabia; wadaan@ksu.sa (M.A.W.); almbaabbad@ksu.edu.sa (A.B.); 6 Department of Chemistry, Karpagam Academy of Higher Education, Coimbatore 641021, Tamil Nadu, India; manojdvrj@gmail.com; 7 Centre for Material Chemistry, Karpagam Academy of Higher Education, Coimbatore 641021, Tamil Nadu, India

**Keywords:** proton exchange membrane, nanomaterial, proton, fuel cell, hydrogen energy

## Abstract

Hydrogen energy is converted to electricity through fuel cells, aided by nanostructured materials. Fuel cell technology is a promising method for utilizing energy sources, ensuring sustainability, and protecting the environment. However, it still faces drawbacks such as high cost, operability, and durability issues. Nanomaterials can address these drawbacks by enhancing catalysts, electrodes, and fuel cell membranes, which play a crucial role in separating hydrogen into protons and electrons. Proton exchange membrane fuel cells (PEMFCs) have gained significant attention in scientific research. The primary objectives are to reduce greenhouse gas emissions, particularly in the automotive industry, and develop cost-effective methods and materials to enhance PEMFC efficiency. We provide a typical yet inclusive review of various types of proton-conducting membranes. In this review article, special focus is given to the distinctive nature of nanomaterial-filled proton-conducting membranes and their essential characteristics, including their structural, dielectric, proton transport, and thermal properties. We provide an overview of the various reported nanomaterials, such as metal oxide, carbon, and polymeric nanomaterials. Additionally, the synthesis methods in situ polymerization, solution casting, electrospinning, and layer-by-layer assembly for proton-conducting membrane preparation were analyzed. In conclusion, the way to implement the desired energy conversion application, such as a fuel cell, using a nanostructured proton-conducting membrane has been demonstrated.

## 1. Introduction

The significance of generating energy from novel and eco-friendly sources has grown in recent years [1]. There are various energy storage sources used to store and release energy, viz., batteries [2,3,4], thermal energy storage [5], hydrogen storage [6], and supercapacitors [7]. Fuel cells are electrochemical devices that, without burning the fuel, which is often hydrogen or hydrogen-rich gases, transform chemical energy from fuel into electrical energy [8]. A technique with tremendous potential for producing power with little harm to the environment is the use of fuel cells [9,10]. Compared to conventional power-generating methods, such as combustion-based generators, they have a number of benefits [11,12]. The advantages include (a) reduced emissions: fuel cells emit a lot less pollution than conventional combustion-based generators, leading to cleaner air and a healthier environment [13]. (b) High electrical efficiency: fuel cells convert a significant portion of the energy from fuel into electricity [14]. (c) Low noise: fuel cells run softly, making them perfect for use in cities or other places where noise pollution is a problem [15]. (d) High dependability: compared to conventional generators, fuel cells have fewer moving components, which lowers the possibility of failure and boosts reliability [16]. (e) Versatility: fuel cells have a variety of uses, including fixed power generation, transportation, and portable electricity [17]. (f) Freedom from fossil fuels: fuel cells can operate on a variety of fuels, such as hydrogen, natural gas, and biogas, reducing our dependence on fossil fuels [18]. (g) Cost-effectiveness: given their high efficiency and low operational expenses, fuel cell systems show promise for long-term cost-effectiveness, despite the fact that their initial expenditures could be significant [19].

Fuel cells function similarly to batteries in that they produce electricity through an electrochemical reaction; however, unlike batteries, they do not require recharging or replacement. Anodes, cathodes, and electrolytes are the typical components of fuel cells, which enable the conversion of fuel into electricity [20]. Fuel cells are a clean and effective way to generate electricity because when hydrogen is utilized as fuel, the reaction only creates heat and water as by-products. Numerous industries [21], including transportation [22], stationary power generation [23], and portable electronics [24], could benefit from the use of fuel cells.

**Figure 1:**
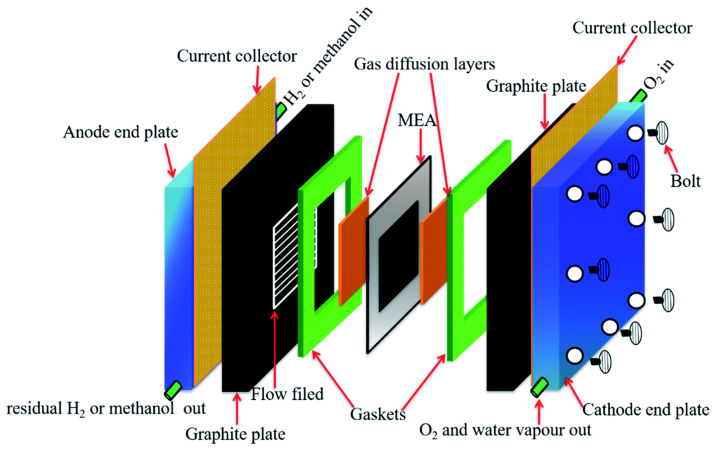
Schematic diagram of operational parts of a single PEMFC. Reprinted with permission from Ref. [26]. Copyright 2021 Royal Society of Chemistry.

Fuel cells, AEM fuel cells, or solid oxide fuel cells employ a proton exchange membrane (PEM), a thin polymer electrolyte membrane, to let protons (hydrogen ions) move more easily between the anode and cathode [25]. The membrane serves as a barrier to keep hydrogen fuel and oxygen from combining with one another, while allowing protons to go back and forth between the anode and cathode compartments of the fuel cell. Figure 1 shows the schematic representation of a single-proton exchange membrane fuel cell (PEMFC). Materials containing perfluorinated sulfonic acids, such as Nafion or other sulfonated polymers, are generally used to create PEMs. They are crucial parts of PEMFCs, which produce electrical energy through the electrochemical reaction between hydrogen and oxygen.

PEM research on fuel cells’ potential for usage in space applications dates back to the early 1960s [27]. Nafion, a product of DuPont, was the only membrane that was known at the time to be capable of conducting protons. It was challenging to use them in fuel cells for extensive commercial applications because of their high cost and scarcity. Other proton-conducting membranes, such as perfluorinated sulfonic acid membranes and sulfonated polyetherketones, were developed by scientists in the late 1960s and early 1970s [28]. These materials were less costly than Nafion and had greater thermal and mechanical stability. Due to its potential for use in fixed power, portable electricity, and mobility, PEM fuel cells attracted greater interest in the 1980s. The performance and durability of PEMs were improved, and low-cost, high-volume production processes were developed. PEMs are now widely employed in many different applications, including fixed power production, backup power, portable electronics, and transportation [29,30]. PEMs and fuel cell systems for clean and efficient energy generation are still being developed as a result of developments in materials science, manufacturing technology, and system engineering [31].

In principle, protons are transported across a PEM, but electrons are prevented from moving through the membrane [32]. A PEM comprises two electrodes, often constructed of platinum or other noble metals, sandwiched between a solid polymer electrolyte. The anode side receives hydrogen gas, which splits into protons and electrons there. The electrons are forced to go via an external circuit in order to reach the cathode, creating an electrical current, while the protons flow through the PEM to reach the cathode side. Water is the only by-product of the reaction between oxygen gas and protons and electrons in the cathode [33]. Protons (H^+^) can be transported through PEMs, which are typically made of a thin, solid polymer sheet that is impermeable to liquids and gas molecules. A perfluorinated sulfonic acid polymer known as Nafion, which has a tetrafluoroethylene (TFE) backbone and pendant sulfonic acid groups, is the most widely utilized substance for PEMs. The proton transport requires ion-conductive channels, which these sulfonic acid groups offer. PEMs have a highly organized structure and a distinct shape made up of hydrophilic and hydrophobic areas. The hydrated clusters of sulfonic acid groups produce hydrophilic domains that aid proton conduction through the membrane. On the other hand, the hydrophobic areas offer mechanical stability and stop fuel and oxidant gases from penetrating the membrane.

The polymerization and subsequent processing of the polymer material are steps in the fabrication of PEMs [34]. The process of copolymerizing monomers, such as TFE and a perfluorovinyl ether monomer with sulfonic acid groups, is the most popular way to create PEMs. The precursor substance is then changed into the proton-conductive form by chemically treating the resultant copolymer. Pre-treatment to ensure the purity of the raw materials, polymerization to make the polymer matrix, and post-treatment to add sulfonic acid groups and enhance ion conductivity are some of the phases in the synthesis process. These procedures are essential for ensuring that the final PEM strikes the ideal balance between proton conductivity, mechanical strength, and chemical stability.

PEMs may be made in a variety of ways, including the solution casting technique: the membrane is created using the solution casting technique by pouring a polymer solution over a substrate and letting it dry [29,35,36]. Although this is a relatively straightforward and inexpensive method, the membrane it produces might not be uniform. Electrospinning: in order to create a membrane, this method uses an electric field to pull polymer fibers out of a solution and deposit them there [37,38]. Although it can take some time, electrospinning creates a membrane that is highly porous and uniform. Sol-gel method: through a series of chemical reactions, a precursor material is first prepared as a solution and then transformed into a solid membrane using this technique [39]. Although they can be customized for particular applications and have a high degree of uniformity, sol-gel membranes can be expensive to produce. In situ polymerization: using this technique, a solvent-assisted chemical reaction between monomers creates the membrane [40,41]. High proton conductivity and great mechanical characteristics may be achieved through in situ polymerization, although reaction conditions can be tricky to manage. Layer-by-layer assembly: using this method, polyelectrolyte layers are alternately deposited to create a membrane [42,43]. Multilayer membranes can be made with fine control over their composition and thickness via layer-by-layer assembly, although the process can be time-consuming and expensive. The approach chosen will depend on the particular application and the desired membrane qualities.

PEMs are suitable for electrochemical applications due to a number of important characteristics. In fuel cells and electrolyzers, a PEM’s main purpose is to make it easier for protons to move between the anode and cathode. Optimal proton conductivity is required for effective device operation. Under the operating circumstances of fuel cells, which frequently involve exposure to acidic or basic environments, high temperatures, and the presence of reactive species, PEMs must demonstrate chemical stability. Long-term stability depends on the sulfonic acid group’s resistance to deterioration and leaching. High temperatures should not cause PEMs to degrade significantly or perform poorly. The membrane’s durability and dependability under diverse working situations are guaranteed by thermal stability. To survive mechanical loads during construction and use, PEMs need to be strong enough mechanically [44]. To preserve their structural integrity, they must be robust yet flexible. In fuel cells, PEMs serve as a barrier to keep reactant gases such as hydrogen and oxygen from mixing. For the membrane to prevent fuel crossover and maintain high cell efficiency, gas molecules must not pass through it. A PEM needs to possess these qualities in order to function properly in electrochemical devices, permitting effective proton transport, chemical stability, and overall device performance.

PEMs, which are used in fuel cells, can function more effectively due in large part to the utilization of nanomaterials [45,46]. A major issue with PEMs is retaining mechanical stability and strength while obtaining high proton conductivity and longevity under demanding working circumstances. These problems can be solved using nanomaterials, which have special qualities that include a large surface area and superior mechanical strength [26,45]. For instance, adding carbon-based nanomaterials to the PEM matrix, such as graphene, carbon nanotubes, and carbon black, can boost proton conductivity by supplying more channels for proton transport [47]. By increasing their resistance to oxidative and chemical degradation, metal oxide nanomaterials, including titanium oxide [48], zirconium oxide [49,50], and cerium oxide [51,52], have also been shown to improve the toughness and stability of PEMs. In order to produce composite PEMs, nanomaterials may also be combined with polymer matrices. This results in a hybrid material with enhanced characteristics. For example, adding titania or silica nanoparticles to a polymer matrix might increase the mechanical stability and strength of PEMs [53]. In general, PEM performance and durability may be greatly enhanced by nanomaterials, and its potential for a range of fuel cell applications is being increased.

Within the scope of this analysis, we focus mainly on the various nanomaterials that are incorporated in PEM for fuel cell applications. This includes metal oxide materials such as silica nanoparticles, titanium dioxide, and zirconium dioxide; carbon-based nanomaterials such as carbon nanotube (CNT) and graphene; and polymer-based materials such as polyhedral oligomeric silsesquioxane (POSS) and polyelectrolyte-functionalized nanoparticles (PEFNs). In addition, various synthesis methods, such as in situ polymerization, solution casting, electrospinning, and layer-by-layer assembly, were reviewed for the nanocomposite of PEMs. Furthermore, various properties of PEM related to the electric, thermal, and structural nature of these modified PEMs are also explored. For this purpose, we have reviewed more than 100 research articles from various databases and research websites such as Google Scholar, ScienceDirect, ACS, etc. This paper consists mainly of six sections; the initial portion deals with the Section 1, which includes the basic principle behind the fuel cells and PEM. The Section 2 elaborates on the findings of using various types of nanomaterials in PEM. Various types of synthesis methods are accessed in the Section 3. Moreover, the properties of PEM, such as electric, thermal, and structural, are included in Section 4. In addition, the future scope of the PEM is discussed in the Section 5, followed by the summary in the Section 6.

## 2. Nanomaterials Doped PEM

Nanomaterials can be used to modify PEMs to achieve a number of advantages, including the following:

- Enhanced Performance: The proton conductivity, mechanical strength, and stability of PEMs treated with nanomaterials have been enhanced. As a result, the fuel cells that employ these PEMs operate more effectively [26,54].
- Cost-effective: Nanomaterials can make PEMs more affordable by lowering the number of expensive components used to create high-performance PEMs [55].
- Durability: PEMs treated with nanomaterials have demonstrated enhanced toughness and resilience to deterioration, making them more appropriate for long-term usage in fuel cells [56].
- High Thermal Stability: PEMs enhanced with nanomaterials have better thermal stability, which makes them more resistant to heat damage, which is crucial for high-temperature PEM fuel cells [57].
- Lower Membrane Crossover: It has been demonstrated that PEMs modified with nanomaterials have lower membrane crossover, which refers to the unintended passage of reactants over the membrane in fuel cells. This increases the effectiveness of the fuel cell and reduces reactant loss [58].
- Compatibility with Different Polymers: Composite PEMs that are compatible with a variety of fuel cell technologies may be created by combining nanomaterials with several kinds of polymers. As a result, the design of fuel cells has more freedom [59].

Overall, the incorporation of nanomaterials into polyelectrolytes offers a viable means of increasing the efficiency, robustness, and affordability of fuel cells.

### 2.1. Metalloids or Metal Oxide-Based Nanomaterials

#### 2.1.1. Silica Nanoparticles

To improve the compatibility between silica nanoparticles (SNP) and polybenzimidazole (PBI) in PBI/SNP nanocomposites, an ozone-mediated process was used to chemically bond PBI to SNPs by Suryani et al. [60]. This process utilized N-(p-carboxyphenyl)maleimide (pCPM)-functionalized SNPs (SNP-pCPM) as precursors. The resulting PBI-functionalized SNPs (SNP-PBI) were characterized and used as inorganic nanofillers to prepare PBI/SNP-PBI membranes for fuel cells. The addition of SNP-PBI to the PBI membranes led to improvements in their thermal and mechanical properties, as well as a decrease in their phosphoric acid uptakes. The PBI/SNP-PBI membrane containing 10% by weight of SNP-PBI exhibited a proton conductivity of approximately 50 mS cm^−1^ at 160 °C, which was 25% higher than that of the pristine PBI membrane. As a result, the PBI/SNP-PBI membrane demonstrated a maximum power density of 650 mW cm^−2^ in a single-cell test, which was higher than the value of 530 mW cm^−2^ obtained from the test for the pristine PBI membrane.

**Figure 2:**
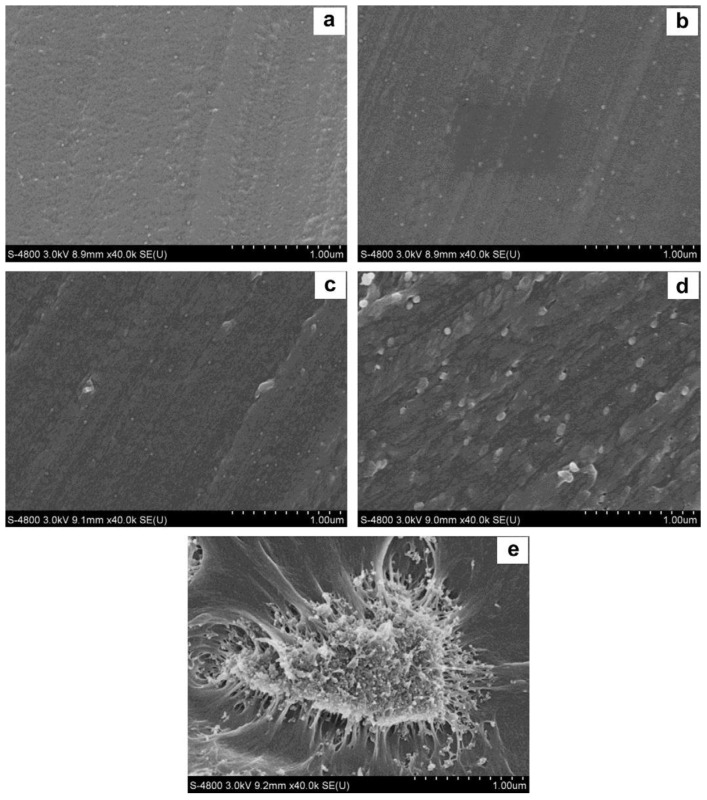
SEM images of (**a**) SPEEK-2.5SSA, (**b**) SPEEK-5SSA, (**c**) SPEEK-7.5SSA, (**d**) SPEEK-10SSA, and (**e**) SPEEK-5SiO_2_. Reprinted with permission from Ref. [61]. Copyright 2012 Elsevier.

Proton exchange membranes were produced by Lin Du et al. by doping sulfonated poly (ether ether ketone) (SPEEK) with varying concentrations of silica sulfuric acid (SSA), which is produced by subjecting SiO_2_ nanoparticles to SO_2_Cl_2_ treatment [61]. This improved both water uptake and conductivity. The uniform distribution of SSA nanoparticles within the resulting composite membranes, as seen in SEM images of the membranes, demonstrates the high level of organic compatibility of SSA particles. Figure 2 illustrates the SEM images of SPEEK-SSA and SPEEK-5SiO_2_.

The distribution of ionic clusters within the composite membranes has improved, as seen in TEM images. In environments with water and low relative humidity, the composite membranes exhibit higher water uptake compared to the pristine SPEEK membrane. Moreover, SSA also significantly improves the composite membranes’ conductivity. These results indicate that SPEEK/SSA composite membranes have the potential to be improved PEMs in PEMFC, with the greatest conductivity of 0.13 S cm^−1^ at 80 °C being demonstrated by the composite membrane containing 5 wt% SSA, which is about 18.6% higher than that of the pristine SPEEK membrane and 8.6% higher than that of Nafion117.

In another work by Yu-Huei Su et al., nanocomposite proton exchange membranes were made with sulfonated sPPEK and then mixed with different concentrations of sulfonated silica nanoparticles (silica-SO_3_H) [62]. The decrease in ion exchange capacity that frequently occurs when non-sulfonated nanofillers are used was mitigated by the use of silica-SO_3_H. Strong –SO_3_H/–SO_3_H bonding was produced as a result of contact between sPPEK chains and silica-SO_3_H particles, which led to an ionic crosslinking effect inside the membrane structure. As a result, both heat stability and methanol resistance increased. The membrane with 7.5 pH of silica-SO_3_H showed little methanol crossover, a large amount of bound water, and a proton conductivity that was 3.6 times higher than that of the membrane made entirely of sPPEK.

The purpose of a study conducted by Jagdeep Kumar Naya and the research group was to determine whether a composite proton exchange membrane composed of sulfonated poly (ether ether ketone), poly (vinylidene fluoride-cohexafluoro propylene), and silicon dioxide (SPEEK/PVdF-HFP/SiO_2_) is suitable for use in microbial fuel cells [63]. To achieve this, different amounts of silica particles (SiO_2_) were added to a mixture of SPEEK/PVdF-HFP. The polymer membrane made of SPEEK (80 wt%) and PVdF-HFP (20 wt%) with SiO_2_-7.5 wt% incorporation had the greatest proton conductivity measurement of 8 × 10^2^ S cm^−1^, as well as the highest voltage production and power density of 998.5 mV and 1.5 mW m^−2^, respectively. In general, research points to the possibility of replacing conventional PEMs in MFCs using SiO_2_-infused polymer composite membranes.

#### 2.1.2. Titanium Dioxide

The development of sulfonated diblock copolymers (SDBCs) for use in PEMFCs that function at low relative humidity (RH) was investigated by Kim, Ae Rhan et al. [64]. The research team produced a hybrid membrane by polycondensing SPEEK and a hydrophobic oligomer, followed by mixing the resultant SDBCs with sintered anatase titanium oxide (S-An-TiO_2_). Subsequently, they created several composite membranes (SDBC/S-An-TiO_2_) with different ratios of S-An-TiO_2_ added to SDBC. The results show that adding S-An-TiO_2_ in the right proportion (i.e., 15 wt%) may considerably improve the composite membrane’s physiochemical characteristics and proton conductivity as well as the PEMFC’s durability and performance under 20% RH. The 1.5 wt% SDBC/S-An-TiO_2_ composite membrane displayed high current and power output, as well as excellent durability values of over 90 h at 60 °C with 20% RH, which were displayed by the 1.5 wt% SDBC/S-An-TiO_2_ composite membrane. These results are believed to be the result of S-An-TiO_2_ and SDBC’s strong interfacial compatibility.

In order to employ a membrane in direct methanol fuel cells, it needed to be able to overcome the drawbacks of proton exchange membranes in acidic conditions and anion exchange membranes in alkaline situations. Sidharthan, K.A. and Joseph, S developed a polyvinyl alcohol-based sodium ion-conducting membrane that can function well in alkaline media [65]. The newly created membrane benefits from both cation exchange membranes and an alkaline medium. The conducting membrane of polyvinyl alcohol–titanium dioxide sodium ion was made by the researchers utilizing a modified phase inversion procedure, which required employing a salt solution as the non-solvent in the coagulation bath. The group evaluated the ionic conductivity, methanol permeability, and V-I properties of the membrane at normal conditions. They evaluated how well the polyvinyl alcohol–titanium dioxide membrane performed in a passive direct methanol fuel cell in comparison to the Nafion 117/Na^+^ membrane and a polyvinyl alcohol-based anion exchange membrane. The results demonstrated that when operating in an alkaline environment under ambient conditions, when using the polyvinyl alcohol–titanium dioxide membrane in the single cell assembly, there was a 17.14% rise in power density in comparison to the Nafion 117/Na^+^ membrane, and a 72.77% increase in comparison to the polyvinyl alcohol-based anion exchange membrane.

Titanium oxide nano tubes (TNT) were synthesized using the hydrothermal process, and their surfaces were subsequently covalently bonded with tungstic acid (an ion exchange group) [66]. High ion exchange characteristics were achieved by fabricating composite membranes with different weight percentages of W-TNT (2%, 4%, 6%, and 8%) and SPEEK. The highest power density of 352 mW cm^−2^ was achieved using an electrolyte in a fuel cell with carbon-supported platinum anode and cathode electrodes at 80 °C. TNT’s hollow structure and extra ion exchange groups (tungstic acid) both played a role in its superior performance over that of a simple membrane.

**Figure 3:**
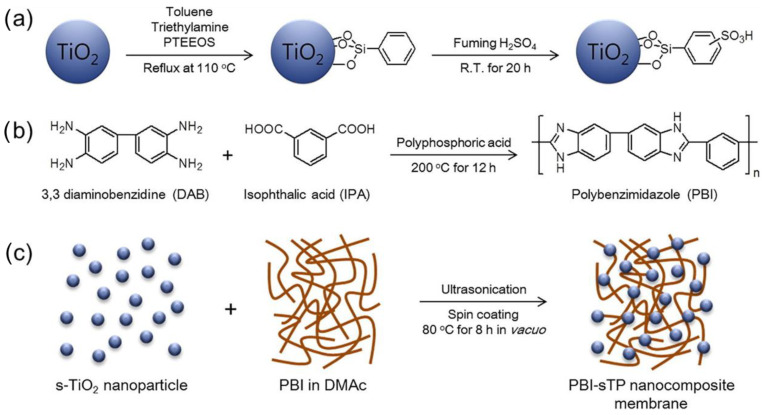
Schematic representation of synthesis of (**a**) the s-TiO_2_, (**b**) meta-PBI, and (**c**) PBI-sTP nanocomposite membrane. Reprinted with permission from Ref. [67]. Copyright 2020 Elsevier.

Sulfophenylated titanium oxide (s-TiO_2_) nanoparticles have been successfully used in a research study to produce high-performance polybenzimidazole (PBI) nanocomposite membranes appropriate for use in HT-PEMFC [67]. Figure 3 denotes the schematic representation of the synthesis of the s-TiO_2_, meta-PBI, and PBI-sTP nanocomposite membrane. The surface of the s-TiO_2_ nanoparticles was altered to yield multifunctional inorganic proton conductors that can boost PBI’s overall performance and proton conductivity. The PBI nanocomposite membranes were doped with phosphoric acid (PA) in order to assess performance. The membranes’ characteristics, such as their proton conductivity, PA retention, and cell performance, were compared to those of clean PBI. According to the findings, PA-doped PBI-sTP2 (2 wt% TiO_2_) had the highest doping level of 12.1 and the lowest proton conductivity at 150 °C at 0.096 S/cm. In the cell test, the peak power density obtained was 621 mW cm^−2^, which is approximately 30% better performance compared to neat PBI (471 mW cm^−2^).

#### 2.1.3. Zirconium Dioxide

**Figure 4:**
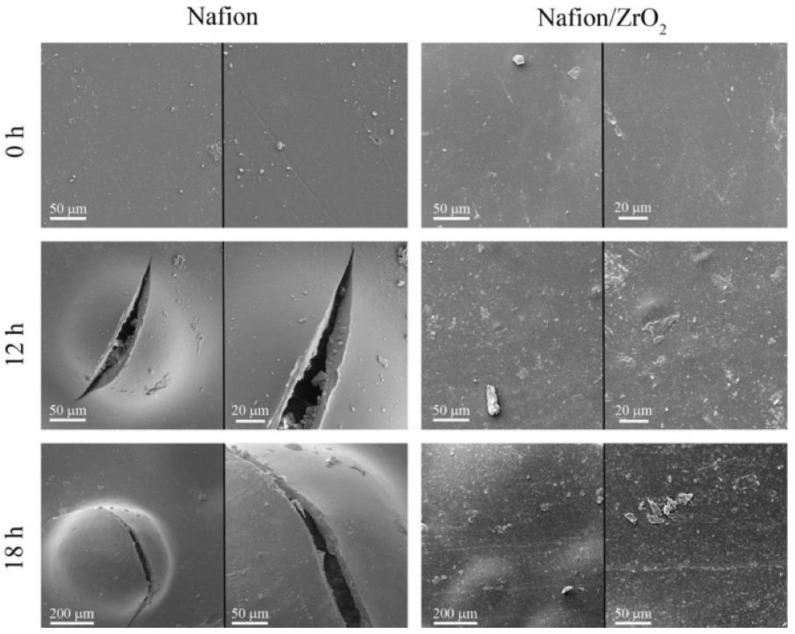
SEM images of pure Nafion and Nafion/ZrO_2_ 4 wt% nanocomposite membranes at different reaction times of Fenton reagent. Reprinted with permission from Ref. [68]. Copyright 2016 Elsevier.

With the use of ultrasonic technology, zirconium dioxide (ZrO_2_) nanoparticles were efficiently produced using the precursors ZrO(NO_3_)2H_2_O, ethylenediamine, and hydrazine [68]. The features of the prepared Nafion/ZrO_2_ nanocomposite membranes were further investigated using measurements of their ion exchange capacity (IEC), proton conductivity, thermal stability, and water absorption. An ex situ Fenton test was used to look into the membranes’ chemical stability. Figure 4 depicts the SEM micrographs of Nafion and Nafion/ZrO_2_ 4 wt% nanocomposite membranes at various Fenton’s reagent reaction times. The findings suggested that the Nafion/ZrO_2_ nanocomposite membrane had superior chemical stability since it showed less fluoride leakage and weight loss than the Nafion membrane alone. 

A proton exchange membrane for a hydrogen/air fuel cell made of a nanofibrous web composite membrane of Aquivion and titanium zirconium oxide (TiO_2_/ZrO_2_) has been developed and tested by Lee, C et al. [69]. Including a small quantity enhanced the hydromechanical stability of the composite membrane (9 wt% membrane) of uniformly dispersed electrospun TiO_2_/ZrO_2_ nanofibrous web. This led to about 2 times higher water retention and 30 times less dimensional change than a pure Aquivion membrane under in-water membrane hydration conditions. At 120 °C and 40% relative humidity, the final composite membrane had a better proton conductivity than the unaltered TiO_2_/ZrO_2_ nanofibrous web composite membrane (0.027 S cm^−1^ versus 0.021 S cm^−1^). In single-cell tests, the Aquivion/TiO_2_/ZrO_2_ nanofibrous web composite membrane outperformed the pure Aquivion membrane in fully humidified conditions (100% RH at 75 and 90 °C), and the single-cell performance was superior in all four testing conditions, with partially humidified high-temperature conditions (120 °C and 40% RH and 140 °C and 20% RH). The outcomes of the accelerated lifespan test demonstrated the composite membrane’s superior durability. Overall, the electrical characteristics and endurance of the fuel cell were improved by the phosphate-modified TiO_2_/ZrO_2_ nanofibrous web composite membrane, particularly at high temperatures (>120 °C).

The study by Mossayebi, Z et al. used sulfated zirconia nanoparticles (SZ) as inorganic additives to improve the characteristics of SPEEK membranes for application in intermediate-temperature fuel cells [70]. Using the response surface method (RSM) and the central composite design (CCD), it was determined how the sulfonation duration and SZ content affected the proton conductivity and oxidative stability of the nanocomposite membranes. The ideal sulfonation period and SZ concentration were determined to be 6.9 h and 5.94 weight percent, respectively. This produced a proton conductivity of 3.88 mS cm^−1^ (at 100 °C and 100% RH) and an oxidative stability of 102 min. Both reactions were more influenced by the sulfonation duration, and the inclusion of SZ nanoparticles enhanced oxidative stability and proton conductivity. 

In this work by Parnian and his group, the Nafion ionomer was synthesized by dissolving the Nafion membrane sources in a high-pressure and high-temperature autoclave reactor [71]. The zirconia nanoparticles were prepared using the microwave-assisted gel combustion technique that was used to construct recast Nafion nanocomposite membranes (R-Nafion/ZrO_2_) with different ZrO_2_ nanoparticle loading. In comparison to the filler-free recast Nafion and commercial Nafion membranes, the nanocomposite membranes showed improved water uptake, mechanical and oxidative stabilities, and increased proton conductivity at all temperatures. The generated nanocomposite membranes are suited for PEMFC applications because the zirconia nanoparticles produced a dense microstructure and excellent nanoparticle dispersion in the membrane matrix.

The investigation by Ketpang, Kriangsak et al. describes a robust and high-performance electrolyte membrane for polymer electrolyte membrane fuel cells operating in low relative humidity [72]. To increase water retention, mesoporous zirconium oxide (ZrO_1.95_) nanotubes (ZrNT) were added to a perfluorosulfonic acid (Nafion) membrane to generate the membrane. By pyrolyzing electrospun zirconium-precursor embedded polymer fibers at 600 °C in an air environment, ZrNT with average diameters of 90 nm was produced. Water transport across the membrane was increased, resulting in a considerable increase in membrane proton conductivity under both completely humid and dry circumstances. This was made possible by the superior water retention and tubular shape of the ZrNT fillers. The Nafion-ZrNT membrane demonstrated a higher power density at 0.6 V at 50% and 100% RH at 80 °C, with increases of 2.7 and 1.2 times, respectively, compared to a commercial Nafion membrane. Under dry circumstances (18% RH at 80 °C), the Nafion-ZrNT membrane also revealed a 3.1 times greater maximum power density than the commercial membrane. The Nafion-ZrNT membrane was also strong, operating for 200 h under 18% RH at 80 °C. When adding mesoporous hygroscopic ZrO_1.95_ nanotubes, ohmic resistance was reduced, improving membrane performance.

### 2.2. Carbon-Based Nanomaterials

#### 2.2.1. CNTs

**Figure 5:**
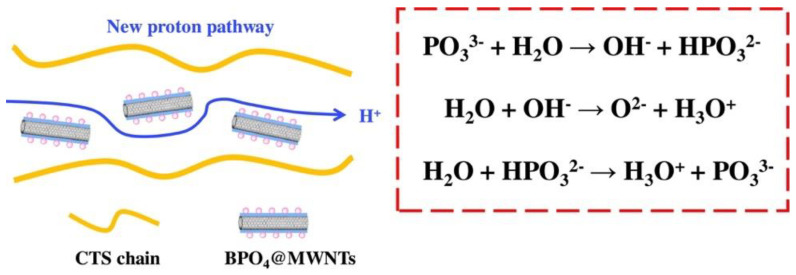
Schematic diagram of proton transport in CTS/BPO_4_@MWNT composite membranes. Reprinted with permission from Ref. [73]. Copyright 2023 Elsevier.

In a research work carried out by Wang, Jie et al., multi-walled carbon nanotubes (MWNTs) were functionalized with super inorganic proton conductor boron phosphate (BPO_4_) using a sol-gel process aided by polydopamine (PDA), producing BPO_4_-coated MWNTs (BPO_4_@MWNTs) [73]. The BPO_4_@MWNTs were then mixed with a weak chitosan (CTS) solution to form a composite membrane made of CTS and BPO_4_@MWNTs. The schematic diagram of proton transport in the CTS/BPO_4_@MWNT composite membranes is shown in Figure 5. The CTS/BPO_4_@MWNT composite membranes have shown excellent outcomes in terms of mechanical property, thermal stability, and oxidation stability owing to the evenly distributed BPO4@MWNT in the CTS matrix and the optimized interface compatibility between the BPO4@MWNT and the CTS matrix. 

The proton conductivity of the CTS/BPO4@MWNTs-2 composite membrane was 0.040 S cm^−1^ at 80 °C and its maximum power density was 49.0 mW cm^−2^ at 70 °C and a concentration of 2 M methanol, both of which were significantly higher than those of the original CTS membranes. Additionally, the BPO_4_ coated on the surface of MWNTs provided an additional proton-conducting pathway.

The development of composite PEMs by fusing metal-organic frameworks (MOFs) with polymeric substrates has attracted a lot of interest in the field of fuel cells. In a study performed by Fu, Jinyu et al., silica-coated carbon nanotubes (SiO_2_@CNTs) and zeolitic imidazolate framework 8 (ZIF-8) precursor were combined to create ZIF-8/silica/carbon (ZSC) material [74]. A variety of SPEEK/ZSC-x composite PEMs were then made using the ZSC material and SPEEK, where x denotes the mass fraction of ZSC and SPEEK. CNTs’ silica covering increased their dispersion in the SPEEK matrix, inhibited direct contact between them, and controlled the nucleation and crystallization of ZIF-8 particles along the CNT shape. Due to the advantageous composition of the ZSC materials and the linked network design, the resultant SPEEK/ZSC composite membranes showed increased performance. The SPEEK/ZSC-0.5 membrane had a proton conductivity of 38.10 mS cm^−1^ at 80 °C, which was 1.8 times more than the conductivity of pure SPEEK (21.12 mS cm^−1^) under the same circumstances. Furthermore, the SPEEK/ZSC-0.5 membrane performed better, with a peak power density in a 2 M methanol solution of 38.9 mW cm^−2^, which was 1.3 times greater than the peak power density of the pure SPEEK membrane (30.3 mW cm^−2^). The composite material maintained good voltage stability even after 100 h of non-stop use at 80 °C, according to stability testing. These findings imply that the fabrication of high-performance PEMs can make use of hybrid materials made of MOFs and functional CNTs.

**Figure 6:**
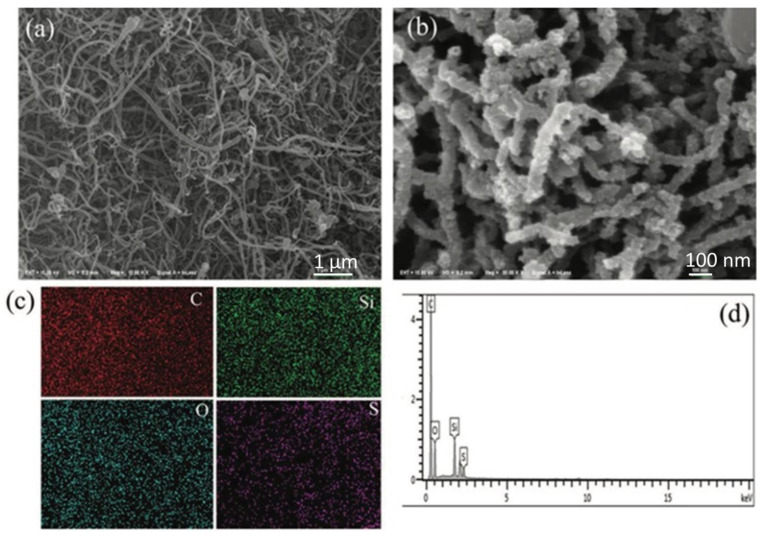
SEM images of (**a**) CNTs and (**b**) SSiO_2_@CNTs, (**c**) EDS mapping, and (**d**) spectrum of SSiO_2_@CNTs from (**b**). Reprinted with permission from Ref. [75]. Copyright 2021 John Wiley and Sons.

CNTs were coated with SiO_2_ using a sol-gel technique in a study by Wu, Silong et al., and then they were grafted with 3-mercaptopropyltrimethoxysilane and oxidized with hydrogen peroxide to produce dual-modified CNTs (SSiO_2_@CNTs) [75]. SSiO_2_@CNT was then added in varied doses to composite proton exchange membranes made of chitosan (CS). Figure 6 shows the SEM images of CNT and SSiO_2_@CNT, the EDS mapping, and the spectrum of SSiO_2_@CNT. Due to their strong compatibility and efficient interface contact with the CS matrix, SSiO_2_@CNTs improved the composite membranes’ thermal stability, mechanical stability, and methanol resistance. Additionally, by producing a large number of transport channels and adding additional proton sources, the inclusion of SSiO_2_@CNTs enhanced the electrochemistry performance. Notably, at 80 °C, the CS/SSiO_2_@CNTs-7 membrane revealed proton conductivity of 35.8 mS cm^−1^ and a tensile strength that was around 1.6 and 2.0 times greater than that of the pure CS membrane, respectively. The membrane also showed a decrease in methanol permeability, measuring 0.9 × 10^6^ cm^−2^ s^−1^. The DMFC performance of the CS/SSiO_2_@CNTs-7 membrane was further enhanced, with a maximum power density of 60.7 mW cm^−2^ at 70 °C and an open circuit voltage of 0.67 V.

Carbon nanotubes (SCNTs) coated with silica were synthesized utilizing the simple sol-gel technique to create chitosan/SCNT (CS/SCNT) composite membranes [76]. The silica layer of the CNTs enhanced the interaction of the SCNTs with the chitosan, resulting in uniform SCNT dispersion in the composite membrane. The silica covering also removed the possibility of electronic short circuiting. However, the lower effective amount of amino functional groups of chitosan caused a reduction in the water absorption of the CS/SCNT membranes. In comparison to the pure CS membrane, the CS/SCNT composite membranes showed superior oxidative and thermal stability, proton conductivity, and mechanical characteristics. According to the study, composite membranes made of CS and SCNTs have a bright future as proton exchange membranes.

Ahmed, Saad et al. aimed to fabricate composite membranes made of chitosan (CS) and multi-walled carbon nanotubes (MWCNTs) for usage in fuel cell applications [77]. To carry this out, 1,3-propane sultone was used to sulfonate MWCNTs, which were subsequently integrated into CS by distillation–precipitation polymerization. According to the findings, CS/MWCNT composite membranes were more thermally and mechanically stable than pure CS membranes. The mobility of the CS chain was restricted by the strong electrostatic contact between the SO_3_H groups of MWCNTs and the NH_2_ groups of CS. In addition, the sulfonated MWCNTs offered effective proton-hopping locations, causing continuous proton-conducting channels to develop. The proton conductivities of the composite membranes with 5 wt% MWCNTs modified by the two distinct techniques were 0.026 and 0.025 S cm^−1^, respectively. According to the study’s findings, fuel-cell applications for CS/MWCNT membranes show potential as PEM.

#### 2.2.2. Graphene

There is a lot of interest in graphene and its derivatives’ possible use in fuel cell technology due to its outstanding physical and chemical properties. The effective separation of protons from hydrogen, which calls for appropriate materials with optimal electrochemical performance, durability, and efficiency, is one of the main problems in fuel cell design. In comparison to conventional membranes, graphene’s flawed structure offers benefits in selectivity, giving scientists and engineers a new, more straightforward method to create fuel cells. Exploring the potential of graphene derivatives in fuel cells has advanced significantly in recent years. This study examines how graphene may be used to improve and apply membrane-based fuel cells. The study shows that graphene materials may operate as active components in membrane-based fuel cells and sheds light on their characteristics, compatibility with other materials, and potential for advancement in the creation of membranes based on graphene derivatives for use in fuel cells in the future.

Through the use of a solution casting technique, sulfonated holey graphene oxide (SHGO) is added to a nanocrystalline cellulose/polyvinyl alcohol (NCC/PVA) matrix by Muhmed, S. A. et al. to create a unique organic–inorganic nanocomposite membrane for polymer electrolyte membranes (PEMs) [78]. Sulfonated graphene oxide (SGO) was created by adding sulfonic acid groups to graphene oxide (GO), and the hole impact that SHGO has on the graphitic plane of GO on NCC/PVA considerably improves the proton conductivity of the membrane. The SHGO encourages proton transport across the membrane by increasing the number of linked proton transfer channels. NCC/PVA-SHGO-1.0 records the greatest proton conductivity values (1.1 × 10^−2^ S cm^−1^) at 80 °C and 100% relative humidity (RH). The hole effect has, nevertheless, caused a modest increase in hydrogen permeability. NCC/PVA’s characteristics and performance have improved with increased SHGO loading. At 80 °C with 100% RH, NCC/PVA-SHGO-1.0 displays the maximum power density and current density of 31.4 mW cm^−2^ and 60.2 mA cm^−2^, respectively. These values are three times greater than those of pure NCC/PVA. Incorporating SHGO into NCC/PVA membranes can greatly enhance their characteristics, functionality, and membrane endurance, according to the study’s findings, making them a promising contender for PEMs.

A nanocomposite polymer electrolyte membrane was designed by Rath, Rosalin et al. using a cost-effective in situ synthesis method, combining graphene oxide and SiO_2_ to create an efficient material [79]. The resulting GS/PVDF-co-HFP nanocomposite membranes were functionalized with chlorosulfonic acid to create SiO_2_-covered graphene oxide nanocomposite membranes (SPCGS) based on sulfonated PVDF-co-HFP (SPCGS), which exhibited improved properties such as increased water absorption, ion exchange capacities, and proton conductivities. The GS nanoparticles in the SPCGS membranes also provided a barrier effect, decreasing methanol permeability due to the tortuosity of the methanol flow channels. The selectivity of the membranes was found to be enhanced up to 3.58 × 10^5^ S cm^−3^, demonstrating their potential in DMFC applications.

Huang, D. and Hwang, J.Y. reported a novel technique for improving PEMs’ functionality for direct methanol fuel cells [80]. The method entailed incorporating hydrocarbon-based PEMs with graphene oxide (GO) that had been dual-functionalized with phosphonic and sulfonic functional groups. The outcomes demonstrated that the PEM’s proton conductivity, ion exchange capacity (IEC), and fuel cell power density were all greatly enhanced by doping functionalized GO. A high IEC value of 2.06 mmol g^−1^, a proton conductivity of 0.084 S cm^−1^, and a power density of 78 mW cm^−2^ were all displayed by the PEM doped with 8% PS-MGO at 25 °C. Additionally, the doped PEM’s methanol permeability was significantly decreased, falling to 18.42 × 10^−7^ cm^2^ S^−1^. According to the study, the effects of dual-functionalized GO were superior to those of single-functionalized GO, and the benefits were directly related to the amount of dual-functionalized GO administered. This improvement was attributed to more acidic sites, which provided more pathways for proton conduction and facilitated proton transportation. Additionally, potential differences between phosphonic and sulfonic groups may have contributed additional energy to push water-connected protons through the membrane beneath the vehicular mechanism. This work offered a potential method for producing PEMs with outstanding IEC, power density, proton conductivity, and methanol permeability for fuel cell applications.

Research work was performed by Theerthagiri, S et al. to determine if titania-graphene-loaded sulfonated polyphenylene sulfone (sPPSS) may be used as a proton exchange membrane in fuel cell applications [81]. The sPPSS nanocomposites were fabricated using the polycondensation process and comprised different concentrations of TiO_2_-GNS (1%, 2%, 3%, and 5%). According to the study, the TiO_2_-GNS dispersed polymer membrane exhibited a lower volume SR (15.6%) and much less water absorption (4.44%) than the control. At 110 °C, the sPPSS nanocomposites with 5 wt% TiO_2_-GNS had a proton conductivity of 2.03 × 10^−2^ S cm^−1^. Additionally, the sPPSS nanocomposites demonstrated excellent OS, with the maximum degradation occurring after 6 h at 80 °C of exposure to the Fenton reagent being only 39.15%. These findings collectively point to the possibility of TiO_2_-GNS distributed sPPSS nanocomposites for usage in PEMFCs.

Devrim, Y. and Durmuş, G.N.B. investigated if polybenzimidazole (PBI)/sulfonated graphene oxide (sGO) membranes are a viable replacement for conventional materials in HT-PEMFC [82]. PBI/sGO composite membranes with various sGO contents were synthesized. Due to the higher concentration of −SO_3_H groups and the accompanying improved channel availability for proton transport, the addition of sGO filler significantly boosted the composite membranes’ proton conductivity. To assess the performance of the composite membranes in high-temperature fuel cells, they were also tested in a single HT-PEMFC. Under non-humid conditions at 160 °C, the composite membrane containing 5 wt% GO (PBI/sGO-2) exhibited the most exceptional performance among the tested membranes, achieving a maximum power density of 364 mW cm^−2^. In comparison, under the same working circumstances, the PBI membrane produced a maximum power density of 235 mW cm^−2^. In order to compare the HT-PEMFC stability of the PBI/sGO composite membranes with the PBI membrane, long-term stability experiments were conducted. The PBI/sGO-2 membrane experienced a performance loss of just 9% during a 200 h performance test, compared to a 13% performance loss for the PBI membrane. These results imply that PBI/sGO composites may have applicability in HT-PEMFC systems.

#### 2.2.3. Fullerenes

The permeability of methanol is a critical issue when using Nafion^®^ membranes as polymer electrolytes in DMFCs. The study by Rambabu G and his group focuses on developing composite membranes for DMFCs by incorporating functionalized fullerene (FF) into Nafion^®^ ionomer at various methanol concentrations [83]. The functionalization of fullerene involved using a precursor called 4-benzene diazonium sulfonic acid, which was obtained through a diazotization reaction with sulfanilic acid. The composite membranes, consisting of Nafion^®^ and FF, were prepared using a solvent casting technique. Notably, the Nafion^®^-FF composite membranes exhibited enhanced proton conductivity attributed to the presence of surface functional –SO_3_H groups. Moreover, these composite membranes displayed improved electrochemical selectivity, resulting in a higher DMFC power output compared to pure Nafion^®^ membranes. Additionally, the optimized composite membrane underwent evaluation for methanol permeability and DMFC polarization at different methanol concentrations. The peak power density achieved in the DMFC reached 146 mW cm^−2^ for the Nafion^®^-FF composite membrane with 1 wt% FF content, using a 2 M methanol solution, surpassing the performance of Nafion-117. Notably, the open circuit voltage of the Nafion^®^-FF composite membrane (1 wt%) exhibited minimal changes over time, confirming its superior stability compared to pure Nafion^®^ and comparable to Nafion-117.

In a groundbreaking study by Tasaki, Ken et al., composite membranes consisting of fullerene and Nafion were successfully fabricated using a novel solution casting method [84]. The fullerenes employed in the composites included C_60_ and polyhydroxy fullerene (PHF), specifically C_60_(OH)_n_ (n~12). Unlike previous methods that relied on doping, the introduction of fullerene into the composite membrane through solution casting led to a more refined dispersion and smaller agglomeration particles. To enhance the miscibility of the hydrophobic fullerene, C_60_, within the Nafion matrix, a new fullerene dispersant poly[tri (ethylene oxide) benzyl] fullerene, denoted as C_60_[CH_2_C_6_H_4_(OCH_2_CH_2_O)_3_OCH_3_]_n_ (n~5), was synthesized in this research. The solution-cast fullerene composites exhibited notable improvements in physical stability, showcasing better integration of the fullerene into the Nafion matrix compared to fullerene-doped composites. Additionally, the solution casting method allowed for higher loadings of fullerene in Nafion, surpassing what was achievable with the previous doping approach. The interactions between the fullerene and Nafion were investigated through ATR FT-IR and molecular dynamics simulations, indicating that PHF predominantly resides in the hydrophobic domain of Nafion at low loading levels. Moreover, voltammetric measurements revealed a reduction in the limiting current density for the fullerene composites compared to Nafion membranes without fullerenes. This work highlights the successful fabrication of fullerene–Nafion composite membranes through solution casting, showcasing improved dispersion, physical stability, and fullerene integration, thereby opening up new possibilities for enhancing the properties of Nafion-based materials.

#### 2.2.4. Nanodiamonds

SPEEK has emerged as a promising alternative to Nafion^®^ membranes for PEMFCs. However, a challenge associated with SPEEK is the trade-off between a high degree of sulfonation (DS) and poor mechanical strength, hindering its widespread application. Hou, Hongying et al. aimed to address this issue by employing two strategies: thermal crosslinking and the addition of nanodiamond particles to the high DS SPEEK matrix [85]. The thermal crosslinking process involved a fraction of the –SO_3_H groups within the SPEEK matrix, forming –SO_2_– bridge bonds to create a three-dimensional (3D) network structure, while the remaining –SO_3_H groups continued to facilitate proton transport. The mechanical and thermal stability of the SPEEK membrane was enhanced through the formation of these crosslinked networks and the interactions between SPEEK and nanodiamonds. Furthermore, the water uptake of the composite membranes showed slight improvement, which could be attributed to capillary condensation occurring within the nanodiamond particles. This investigation highlights the successful implementation of a dual approach involving thermal crosslinking and nanodiamond incorporation to overcome the challenge of balancing DS and mechanical strength in SPEEK membranes, thereby enhancing their potential for use in PEMFC applications.

In another work by Postnov, V. N. et al., the impedance spectroscopy method was utilized to investigate composite solid polyelectrolytes based on Nafion and Aquivion, which incorporated deagglomerated detonation nanodiamonds [86]. The study revealed that the inclusion of these dopants resulted in a significant enhancement in the proton conductivity of the hybrid membranes, particularly under low relative humidity conditions.

A novel approach was employed to enhance the proton conductivity mechanism in perfluorinated membranes for hydrogen fuel cells [87]. The strategy involved modifying the composite short-side chain membranes by incorporating functionalized diamond nanoparticles. By embedding carboxylated nanodiamonds into the polymer matrix, the conductivity of the membranes experienced a noticeable increase, even at moderate nanodiamond concentrations, while maintaining a high level of mechanical strength. The membranes were prepared using a casting method from solutions in N,N-dimethylformamide, which enabled the preservation of the overall channel structure in the presence of nanodiamonds, as confirmed by neutron scattering data. The proposed mechanism suggests the formation of additional conductive regions controllably generated by the association of nanodiamond particles with polymer chains. These regions promote accelerated proton diffusion through the hopping Grotthus mechanism, originating from proton adsorption centers. This diffusion occurs on the surface of the polymer chains, ultimately transitioning to proton-conducting channels within the matrix that are covered with sulfonic groups. Overall, this review highlights the successful application of modifying perfluorinated membranes with functionalized diamond nanoparticles, leading to improved proton conductivity by establishing additional conductive regions and maintaining membrane integrity.

Compositional membranes that facilitate the conduction of protons, utilizing perfluorinated copolymers of the Aquivion^®^ type and incorporating detonation nanodiamonds (DND) with positively charged surfaces, were developed with the aim of enhancing the performance of hydrogen fuel cells [88]. Through small-angle neutron scattering (SANS) experiments, it was observed that these membranes, filled with DND at concentrations ranging from 0% to 5% by weight, maintained the characteristic conducting channels found in Aquivion^®^ membranes, while the submicron-scale polymer domains were adorned with DND particles measuring approximately 4–5 nm in size, as confirmed by scanning electron microscopy (SEM) analysis. Furthermore, membrane-electrode assemblies (MEAs) operating in the O_2_/H_2_ system were employed to assess the modified membranes with different compositions. The results exhibited several improved functional properties compared to pristine membranes without additives, including enhanced operational stability, reduced proton resistance, and higher current densities at elevated temperatures within the extended temperature range of 22–120 °C.

### 2.3. Polymeric Nanomaterials

**Figure 7:**
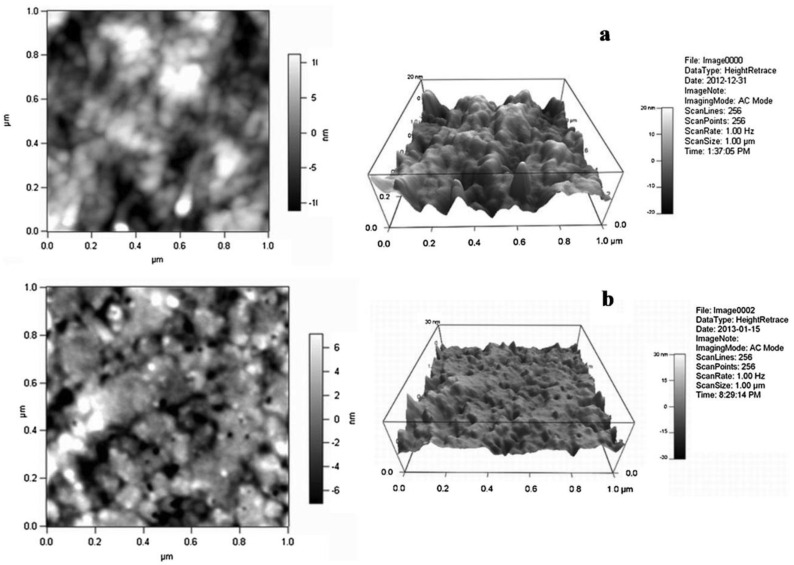
AFM images of (**a**) POSS-(PMMA26-b-SPS156)8 and (**b**) POSS-(PMMA16-b-SPS200)8. Reprinted with permission from Ref. [89]. Copyright 2015 Elsevier.

Using atom transfer radical polymerization (ATRP), star-shaped block copolymers containing POSS were synthesized by Zhang, Jie et al. [89]. The polymerization of polymethyl methacrylate (PMMA), the first building block, and polystyrene (PS), the second building block, was started using the POSS-(Cl)8 initiator. Star-shaped ionic polymers (POSS-(PMMA-b-SPS)8) were created by sulfonating the polystyrene block and were tested as proton exchange membranes (PEMs). Figure 7 demonstrates the AFM images of POSS-(PMMA26-b-SPS156)8 and POSS-(PMMA16-b-SPS200)8. The PEM with a longer SPS block had better proton conductivity at low relative humidities (RHs) than the PEM with a shorter SPS block under the same hydration number circumstances. This was caused by the former’s hydrophilic domains being more closely linked, as seen by electron microscopy. At 100% RH, however, the conductivity trend for the two PEMs changed. 

The inverted conductivity pattern was explained by low-field nuclear magnetic resonance analysis, which revealed that the shorter SPS block PEM contained more loosely bound water than the longer SPS block PEM at 100% RH. This work reveals that in order to obtain high proton conductivities in PEMs, it is crucial to consider both ionic domain structure and water–polymer interaction.

For PEMFC to have a high power density and long-term durability, proton exchange membranes with high proton conductivity and stability must be developed. Proton conductivity and swelling ratio, however, frequently have to be traded off in PEMs made from sulfonated poly (arylene ether sulfone) (SPAES). To solve this problem, a series of SPAES composite membranes were made using a UV radiation technique and a gradient distribution of POSS (polyhedral oligomeric silsesquioxane) nanospheres by Chen, F et al. [90]. Under fully hydrated conditions at 80 °C for POSS/S50, the numerous POSS nanospheres function as reactive interfacial modifiers, and their hydrophobic moieties are immobilized in an orderly manner into the SPAES matrix, resulting in an extremely low swelling ratio (1.0%) and high proton conductivity (0.09 S cm^−1^). PEMFCs and other functional membrane-related research, including batteries and filtration, will be significantly impacted by this novel method for producing large-size composite membranes with a gradient structure.

Using aminopropyloligosiloxane (NH_2_-POSS) as a molecular hybrid filler, a non-humidified SP/IL/NH_2_-POSS hybrid membrane and a self-assembled medium temperature were developed by Guan, Z and co-workers in order to increase the conductivity of SPEEK/SP membranes and stop the loss of ionic liquid (IL) [91]. In order to create proton transport channels, NH_2_-POSS was assembled into the hydrophilic domain ion phase of SPEEK, where the –NH_2_ of NH_2_-POSS and the –SO_3_H of SPEEK formed acid-base pairs. Additionally, IL was retained, and conductivity was synergistically increased thanks to NH_2_-POSS’ enrichment of IL in the hydrophilic domain. The SP/IL/NH_2_-POSS-2 membrane exhibited a conductivity of 5.51 mS cm^−1^ at 200 °C without humidification, which was around four times that of the SP/IL membrane, and an IL loss rate of 46.9 wt%, which was nearly 55.8% that of the SP/IL membrane.

Nanomaterials are also used in anion-exchange membranes, such as N-methylpiperidium functionalized polyhedral oligomeric silsesquioxane developed by Sang, J and team to improve the mechanical, thermal, and ion conductivity of anion-exchange membranes based on styrene–ethylene/butadiene–styrene [92]. 

## 3. Synthesis Methods of Nanomaterials Modified PEM

There are several preparation methods available for incorporating nanomaterials into proton exchange membranes. Some of the commonly used methods are as follows:

- In situ polymerization: To obtain a copolymer, the polymerization process is carried out either with or without additives, leading to the formation of a composite. When monomers and nanoadditives are present during synthesis, the resulting copolymer incorporates the nanoadditives. Following the polymerization process, the product undergoes purification, and subsequent drying yields a polymer powder. To prepare a membrane, a solution is typically prepared from the polymer powder, and then casting techniques are employed.
- Solution casting: In this method, the nanomaterial is first dispersed in a solvent, and then the polymer solution is added to it. The resulting mixture is then cast into a thin film and dried to form the nanocomposite membrane.
- Electrospinning: In this method, a polymer solution containing the nanomaterial is electrospun into a nanofiber membrane. The nanofiber membrane has a high surface area and excellent mechanical strength.
- Inclusion of nanomaterials in the pre-formed membrane: This method involves the incorporation of nanomaterials into pre-formed proton exchange membranes. The nanomaterials can be added by impregnation, in situ growth, or coating.
- Layer-by-layer assembly: This method involves the layer-by-layer deposition of nanomaterials and polyelectrolytes to form a nanocomposite membrane. The resulting membrane has a well-defined structure and high proton conductivity.

### 3.1. In Situ Polymerization

The 3,3′-disulfonate-4,4′-dicarboxylbiphenyl (DSBP), 4,4′-dicarboxylic diphenyl ether (DCPE), and 2,5-diamino-1,4-benzenedithiol dihydrochloride (DABDT) were directly polycondensed to produce a series of sulfonated polybenzothiazoles (sPBT-E) by Wang, G et al. [93]. Due to the flexible ether groups in their backbone, the sPBT-E polymers demonstrated significant solubility in common organic solvents. For the first time, they doped sulfonated graphene oxide (SGO) into the sPBT-E matrix with a sulfonated monomer ratio of 62.5% (referred to as sPBT-E62.5/SGO) in order to create composite proton exchange membranes (PEMs). For comparison, they also made sPBT-E membranes with various sulfonated monomer ratios (55, 57.5, 60, and 62.5). The membranes of the sPBT-E and sPBT-E62.5/SGO series both demonstrated outstanding dimensional stability, suitable water uptake, strong thermal stabilities, and mechanical qualities. The inclusion of SGO’s ion-transporting sites led to continuous proton transfer channels that improved the sPBT-E62.5/SGO membranes’ proton conduction. Thus, the proton conductivity and power density of sPBT-E62.5/SGO3 were up to 0.139 S cm^−1^ and 519.9 mW cm^−2^, respectively, at 80 °C and 100% relative humidity (RH), which were better than those of the sPBT-E series and Nafion 212 (0.133 S cm^−1^, 413.2 mW cm^−2^). The output voltage of the sPBT-E62.5/SGO3 membrane only dropped by 13.2% after 20 h of non-stop use, which was less than the 22.7% drop of the sPBT-E62.5 membrane. The sPBT-E62.5/SGO3 membrane has therefore shown potential as a substitute PEM for PEMFCs.

In the study of Lin, Bencai et al., for the development of high-temperature hybrid proton exchange membranes (PEMs), graphene oxide functionalized with 1-(3-aminopropyl)-3-methylimidazolium bromide ([APMIm][Br]-GO] and 1-methylimidazolium trifluoromethanesulfonate ([MIm][TfO]) were used as fillers and proton carriers, respectively [94]. The protic ionic liquid (PIL, [MIm][TfO]), polymerizable oils (styrene/acrylonitrile and divinylbenzene), and various concentrations of [APMIm][Br]-GO were combined to create the PEMs. The membranes have remarkable mechanical and thermal stability qualities. The proton conductivity of the hybrid membranes is significantly increased by the integration of [APMIm][Br]-GO, with the membrane containing 1.0 wt% [APMIm][Br]-GO exhibiting the maximum conductivity (up to 1.48 × 10^−2^ S cm^−1^ at 160 °C). The hybrid membranes containing [APMIm][Br]-GO demonstrate substantially superior PIL retention capabilities than the ordinary membrane without it. The PIL-based hybrid membranes are appropriate for high-temperature PEMFC applications due to these characteristics. 

**Figure 8:**
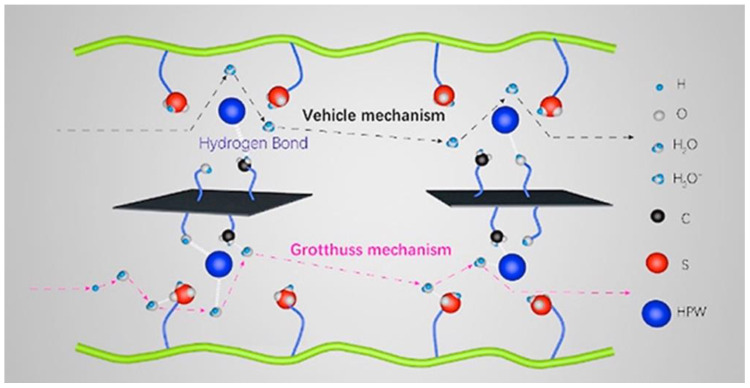
Schematic diagram illustrating Grotthuss and Vehicle processes. Reprinted with permission from Ref. [95]. Copyright 2020 Elsevier.

Using chloromethylated sulfonated polyether etherketone (SPEEK-Cl), Friedel-Crafts alkylation at 130 °C was used in a work by Sun, F et al. to develop a self-crosslinked sulfonated polyether etherketone membrane (C-SPEEK) [95]. With a decreased water uptake (WU) of 47.75% and swelling rate (SR) of 8.69% at 60 °C, C-SPEEK demonstrated superior dimensional stability and proton conductivity in comparison to the SPEEK. The composite membranes were doped with carbon nanomaterials (reduced graphene oxide, rGO; graphite oxide, GO; and carbon nanotubes, CNTs) and phosphotungstic acid (HPW) to further improve proton conduction. Improved conductivities were obtained as a result of the inclusion of HPW and carbon nanomaterials, which promoted proton conduction via both the Grotthuss and Vehicle processes. Figure 8 illustrates the schematic diagram of the Grotthuss and Vehicle processes.

The proton conductivity of C-SPEEK/HPW/GO at 80 °C was 119.04 mS cm^−1^, which was 2.4 times more than that of C-SPEEK. Additionally, in H_2_/O_2_ single-cell tests, C-SPEEK/HPW/GO demonstrated superior performance with a higher power output of 876.80 mW cm^−2^, as opposed to C-SPEEK’s 776.72 mW cm^−2^. These findings imply that C-SPEEK/HPW/GO and C-SPEEK are viable options for fuel cell applications as membrane materials.

Yu, DM et al. reported the synthesis of functionalized zeolite utilizing 1,3-propane sultone as a sulfonic acid functionalization agent in order to improve the proton transport characteristics for high-temperature (120 °C) PEMFCs [96]. To carry this out, sulfonated poly (arylene ether sulfone) (SPAES) copolymers were combined in different ratios with sulfonated zeolite to create composite membranes. The sulfonated zeolite was predicted to enhance mechanical characteristics and proton conductivity. The study made use of the SPAES copolymer, which was created by direct polymerization and had a 50 °C sulfonation. At 120 °C and 50% relative humidity, the composite membrane containing 5 wt% sulfonated zeolite showed an increase in proton conductivity from 0.022 S cm^−1^ to 0.030 S cm^−1^. The synthesized composite membrane with 5 wt% sulfonated zeolite demonstrated an impressive 54% increase in power density in the single-cell test, making it a potential material for high-temperature PEMFC applications.

Yang, Tianjian et al. proposed a covalent cross-linking of functional polymer brush-modified graphene oxide (FPGO) to sulfonated polysulfone (SPSU) matrix, which resulted in the synthesis of a unique cross-linked nanocomposite membrane [97]. Using functional linear polysiloxane brushes, surface precipitation polymerization and chemical modification were used to create the FPGO. A pure SPSU membrane and SPSU/GO composite membranes were used to create control groups. Reduced inorganic filler agglomeration and better interfacial contact in the SPSU/FPGO cross-linked membranes increased the membrane’s resistance to methanol diffusion and produced continuous channels for rapid proton transportation at high temperatures. The membranes also showed enhanced stability and strong mechanical performance, guaranteeing long-lasting proton conducting. Among all the prepared membranes, including Nafion^®^117, the SPSU/FPGO-1 cross-linked membrane displayed the best overall properties, having a low methanol permeability of 1.71 × 10^−6^ cm^2^ s^−1^ at 30 °C and a proton conductivity of 0.462 S cm^−1^ at 90 °C under hydrated conditions. The SPSU/FPGO cross-linked membrane’s potential for use in DMFCs was proved by the remarkable membrane selectivity.

In a work reported by Peng KJ and team, atom transfer radical polymerization (ATRP) employing Nafion chains as macroinitiators were used to develop a copolymer called poly (styrenesulfonic acid)-grafted Nafion (Nafion-g-PSSA) [98]. The ATRP of the styrenic monomer is started by the C–F connections of the –CF– groups and –CF_2_–SO_3_H groups in the Nafion chains. In comparison to the pure Nafion membrane, the Nafion-g-PSSA membrane has larger ionic cluster domains and shorter distances between the domains because the PSSA chains enhance the microphase separation between the hydrophilic and hydrophobic domains. As a result, Nafion-g-PSSA is a useful addition for proton exchange membranes based on Nafion. The improved Nafion membrane with a 15 wt% optimum fraction in comparison to a commercial Nafion 212 membrane (88 mS cm^−1^) and a recast neat Nafion membrane (53 mS cm^−1^), Nafion-g-PSSA has a higher proton conductivity of about 97 mS cm^−1^ (95 °C, RH = 100%). An effective approach to developing Nafion-based proton exchange membrane materials is proved as the Nafion-initiated ATRP method.

### 3.2. Solution Casting

Ceria nanoparticles can be used to increase the toughness of SPEEK membranes for fuel cell applications. A study by Parnian MJ et al. looked at how well and how long SPEEK/ceria nanocomposite membranes with various ceria loadings performed [99]. Utilizing a microwave-assisted gel combustion process, ceria nanoparticles were obtained. The solution-casting technique was used to synthesize the SPEEK/ceria nanocomposite membranes. The nanocomposite membrane has better qualities than pure SPEEK membranes, according to the physical, thermal, and chemical assessments. The SPEEK nanocomposite membranes demonstrated better chemical stability, which was proven by proton conductivity measurements taken before and after a 100 h Fenton test. Polarization curves at various temperatures were used to assess the performance of the fuel cell, and accelerated stress tests were carried out by keeping the cell at open circuit voltage at low humidity for 230 h. The breakdown rate of the nanocomposite membranes was lower than that of the pure SPEEK membrane, and it got slower as the ceria concentration rose. The SPEEK nanocomposite membranes were extremely stable, as evidenced by the gas crossover, polarization curves, and ohmic resistance measurements performed pre and post-accelerated stress testing. The findings imply that attractive possibilities for proton exchange membrane fuel cells include SPEEK/ceria nanocomposite membranes.

**Figure 9:**
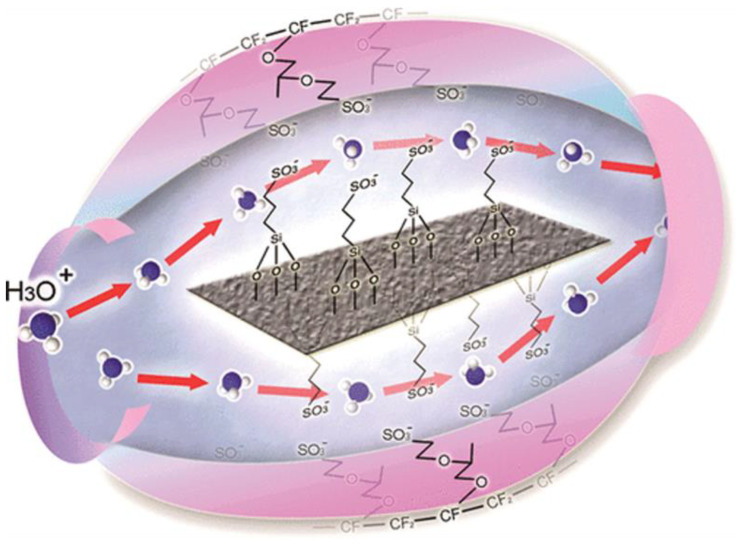
Schematic representation of the F-GO/Nafion composite membranes by solution casting. Reprinted with permission from Ref. [100]. Copyright 2011 American Chemical Society.

Functionalized graphene oxide Nafion nanocomposites (F-GO/Nafion) are suggested as a possible substitute for high-temperature PEMs in fuel cell applications. In a work by Zarrin H et al., natural graphite flakes were treated using the modified Hummer’s technique to make the GO nanosheets, and 3-mercaptopropyl trimethoxysilane (MPTMS) functionalization was used to add sulfonic acid functional groups [100]. Simple solution casting was used for making the F-GO/Nafion composite membranes. Figure 9 depicts the formation of F-GO/Nafion composite membranes by solution casting. At 120 °C with 25% humidity, proton conductivity and single-cell testing showed that F-GO/Nafion membranes perform four times better than recast Nafion, which is a substantial improvement.

Using an organo-soluble, fluorine-containing PBI copolymer, tetraethoxysilane (TEOS) as the silica precursor, and a bonding agent, new polybenzimidazole (PBI)/silica nanocomposite membranes were made by Chuang SW et al. [101]. The bonding substance enhanced the silica nanoparticles’ ability to engage with PBI chains at the interface. The PBI membranes’ thermos oxidative stability somewhat improved as a result of the silica addition, and the nanocomposite membranes’ coefficients of thermal expansion (CTEs) decreased as the silica level rose. The inclusion of silica also improved the PBI films’ mechanical characteristics and methanol barrier capacity. When compared to pure PBI films, the modulus of the PBI/10 wt% silica nanocomposite membranes increased by 37%, and the permeability to methanol decreased by 58%. The results suggested that the PBI/silica nanocomposite membranes have potential applications in fuel cells due to their improved mechanical properties and methanol barrier ability, despite the fact that the conductivities of the acid-doped PBI/silica nanocomposites were marginally lower than those of the acid-doped pure PBI. 

It was reported by Vani R and co-workers that PEMFCs can employ PVA membranes that have been crosslinked with sulfonated carbon nanotube (SCNT) fillers using a solution casting technique [102]. Due to their increased ionic conductivity and mechanical robustness, SCNTs are well recognized to be very efficient additions to ionic membranes. It has been discovered that even very tiny SCNT concentrations (0.01 wt%, 0.1 wt%, 0.5 wt%, and 1 wt%) significantly affect the membrane characteristics. The membranes’ ability to absorb water, their ability to exchange ions, and their mechanical strength were all tested. PVA-SSA-0.1 wt% SCNT demonstrated the best power density of all compositions evaluated, while PVA-SSA-0.5 wt% SCNT showed the least susceptibility to variations in reactant humidity during short-term performance. In contrast, PVA-SSA-0.1 wt% SCNT displayed 0.21 W cm^−2^ at 0.4 V, whereas Nafion^®^115 generated 0.33 W cm^−2^ at this voltage. The polarization curve was adjusted to account for the overpotential associated with hydrogen gas purity, which led to a considerably increased power density of 0.4 W cm^−2^. 

In order to create cross-linked nanocomposite membranes with better chemical, thermal, and mechanical durability, the study by Beydaghi H and group combined aryl sulfonated graphene oxide (SGO) with poly (vinyl alcohol) [103]. The cross-linking technique was used to increase the stability of the nanocomposite. The graphene oxide nanoparticles’ surfaces were altered using an aryl diazonium salt of sulfanilic acid. The thermal stability (melting temperature, Tm = 223 °C), mechanical stability (tensile strength, TS = 67.8 MPa), and proton conductivity (=0.050 S cm^−1^) of the nanocomposite proton exchange membranes were all improved by adding SGO to the PVA matrix (at 5 wt%). A proton exchange membrane fuel cell (PEMFC) with a maximum power density of 16.15 mW cm^−2^ at 30 °C was obtained using the PVA/SGO membrane. The findings imply that PVA/SGO nanocomposite membranes have application potential in PEMFCs.

### 3.3. Electrospinning

The use of reinforced composite membranes (RCMs), which combine porous polytetrafluoroethylene (PTFE) as a mechanical reinforcement and perfluorosulfonic acid (PFSA) as a proton conductive polymer, has shown great promise for enhancing the performance of polymer electrolyte membrane fuel cells. The hydrophilic sulfonate groups in PFSA and the hydrophobic nanoporous PTFE matrix interact in an incompatible manner, which makes it difficult to generate the polymeric nanocomposites needed for RCMs. Hwang CK and his research group have reported a straightforward and sophisticated manufacturing method for making a cross-aligned PTFE (CA-PTFE) framework [104]. This included annealing electrospun conjugated polymers to produce a distinctive micron-scale grid-type PTFE matrix using electric-field guided electrospinning. The CA-PTFE RCM’s resultant continuous fibrous structure of PTFE particles is impregnated with PFSA, enabling effective proton transport through the concentrated sulfonate groups while reducing swelling and fostering membrane hydration. The CA-PTFE RCMs demonstrated exceptional fuel cell performance in both low and high humidity operation, with a maximum power density of 0.85 W cm^−2^ at 100% RH conditions and a current density of 1.38 A cm^−2^ at 0.6 V. The CA-PTFE RCM also showed extremely reduced hydrogen crossover (less than 5 mA cm^−2^ at 0.4 V) and long-lasting single-cell operation after 21,000 wet/dry cycles, exceeding the membrane strength requirements for transportation applications. Fibrous PTFE reinforcements’ rational design opens up new possibilities for creating extremely stable PEM fuel cells.

**Figure 10:**
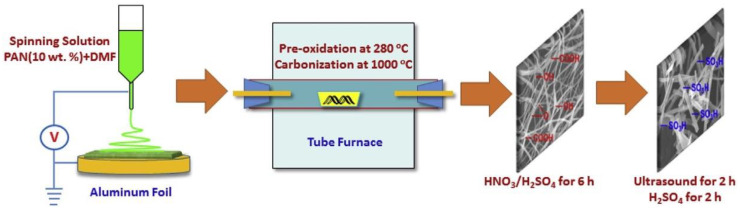
Method for the synthesis of SCNFs. Reprinted with permission from Ref. [105]. Copyright 2017 Elsevier.

Building a good polymer/inorganic filler interface at the microstructural level is required to design high-performance proton-conducting materials in organic/inorganic composite proton exchange membranes. This was accomplished by Liu X et al. through electrospinning carbon nanofibers (CNFs) and then sulfonating them to develop interfacial compatibility with SPEEK via hydrogen bonding interactions [105]. Figure 10 indicates the method for the synthesis of SCNFs. It was shown that doping with SCNFs of different compositions greatly improved the physical characteristics of the composite membranes, especially in terms of proton conductivity, mechanical strength, and methanol permeability.

Overall, this work offers insightful information on creating composite membranes based on SCNF for fuel cell applications.

In direct methanol fuel cells (DMFCs), methanol crossover is a serious issue, and surface modification has come to light as a viable remedy. Awang N et al. developed an electrospun nanocomposite membrane with an exfoliated morphology with cloisite and SPEEK [106]. Electrospun SPEEK/cloisite fiber mats were submerged in a SPEEK polymer matrix that had partially hardened to create the membrane. With a current density of 7.73 mA cm^−2^ and a power density of 1.18 mW cm^−2^, the results showed that the completely exfoliated configuration of SP/e-spunCL15 outperformed other membranes, including Nafion^®^117. The electrospinning method was discovered to create a well-dispersed cloisite15A, which boosted the membrane’s performance for direct methanol fuel cell applications by improving the membrane’s morphological and dimensional features.

Although the use of nanofiber-based ion-exchange membranes in energy applications has some special benefits, controlling the bead formation and diameter of nanofibers during the electrospinning process is crucial and difficult. Mohammadi M et al. proposed the synthesis and characterization of 40% sulfonated hydroquinone-based polysulfones [107]. Experiments were constructed using the Taguchi approach using polymer solution concentration, voltage, feed rate, and needle-to-collector distance as design factors to examine the effects of electrospinning parameters on nanofiber shape and diameter. To reduce the diameter, genetic algorithm optimization was employed. The most important factor, according to the results, was the concentration of the polymer solution, which led to the creation of thin, bead-free nanofibers with an average diameter of about 97.5 nm. Additionally, the conductivity measurements of the nanofibrous mat were higher (0.1105–0.2851 S cm^−1^) than those of dense membranes (0.0791–0.2201 S cm^−1^) in the water temperature range of 20–100 °C.

The movement of protons is necessary for the operation of important energy conversion mechanisms in biological processes. Using biofunctional SiO_2_ nanofibers integrated into a Nafion matrix, Wang H and his co-researchers produced the innovative bioinspired Nafion membrane known as Bio-Nafion [108]. The SiO_2_ nanofibers were made by electrospinning tetraethyl orthosilicate-derived silica sol, and they were further functionalized with the amino acids cysteine, serine, lysine, and glycine to form effective proton transport channels that featured plenty of H^+^ transport sites. The composite membranes made of Nafion-Cys, Nafion-Lys, Nafion-Ser, and Nafion-Gly were synthesized by oxidizing the cysteine-functionalized SiO_2_ nanofibers. On a number of membrane properties, including water uptake, thermal stability, dimensional stability, methanol permeability, and proton conductivity, the research team assessed the effects of the various polar groups (SO_3_H, OH, and NH_2_) of the amino acids. Due to glycine’s lesser amount of hydrophilic groups when compared to other amino acids, Nafion-Gly had the lowest proton conductivity and water absorption, with a value of 0.2424 S cm^−1^ (80 °C). Overall, the proton conductivity, dimensional stability, and methanol permeability of the composite membranes were greatly improved by the addition of Bio-SiO_2_ nanofibers.

### 3.4. Layer-by-Layer Assembly

**Figure 11:**
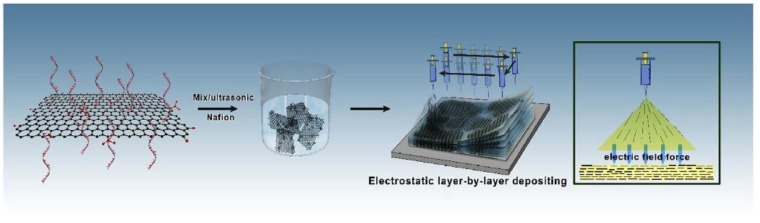
Schematic representation of the PEM synthesis with ordered DNA@GO nanosheets via ELD. Reprinted with permission from Ref. [109]. Copyright 2020 Elsevier.

Using a novel method of building proton transport channels, Wang H et al. were able to synthesize a high-performance PEM [109]. A Nafion matrix and single-strand DNA (ss-DNA) are functionalized with ordered graphene oxide (GO), which is electrostatically deposited layer by layer to create the membrane. Figure 11 illustrates the schematic representation of the PEM synthesis with ordered DNA@GO nanosheets via ELD. The impacts of various ss-DNA@GO content ratios on the composite PEMs’ proton conductivity, methanol permeability, and single-cell performance were studied. The resultant composite membrane exhibits a low methanol permeability of 1.63 × 10^−7^ cm^2^ s^−1^ at ambient temperature and a high proton conductivity of 351.8 mS cm^−1^ at 80 °C and 100% RH.

Furthermore, the team put the composite membranes together into direct methanol fuel cells and discovered that Nafion/DNA@GO-2/5 showed a maximum power density of 255.33 mW cm^−2^ at 60 °C, which is 2.42 times higher than pure Nafion. This research shows the potential of DNA@GO in PEMs and offers a bio-inspired method for building high-performance PEMs in the future for fuel cells.

Jia T et al. used the layer-by-layer (LBL) self-assembly method to incorporate carbon nanotube (CNT) and CNT-based inorganics into polymer systems of polyurethane (PU) and chitosan (CS) in order to produce high-temperature PEMs [110]. By alternately depositing PU as polycations and CS and inorganic materials based on CNTs as polyanions, these ultrathin and conductive membranes were made. The membranes were subsequently coupled with phosphoric acid (PA) molecules via intermolecular hydrogen bonding to create PA-doped membranes. The LBL membranes’ multilayered structure increased the proton conductivity of PA-doped membranes and decreased the impedance to proton conduction. At 150 °C and an activation energy of 22.9 kJ mol^−1^, the (PU/CNT-CdTe/PU/CS)150/60%PA membranes had the maximum proton conductivity of 6.82 × 10^2^ S cm^−1^. The research implies that LBL self-assembly is a potential method for creating ultrathin and layered membrane electrolytes, and CNT-based inorganics can serve as effective proton carriers for high-temperature PEMs.

A well-liked approach for producing layered membranes at the nanoscale, LBL self-assembly has applications in bionics, renewable energy, and microelectronics. The goal of the work performed by Jia T et al. was to create multilayered membranes with ordered deposition of components using the LBL self-assembly approach [111]. By identifying an ordered component distribution and a compact structure, the successful synthesis of anhydrous PEMs based on poly (diallyl dimethyl ammonium chloride) (PDDA) as polycations, graphene oxide (GO) as polyanions, and polyurethane (PU) as polyanions were demonstrated. These membranes interacted with PA molecules when submerged in phosphoric acid (PA) solution to create PA-doped membranes. Although PA predominated proton conduction, the multilayered structure led to a lower activation energy and proton conduction resistance. Proton conductivity for the (PU/GO/PDDA/GO)200/60%PA membranes was 1.83 × 10^−1^ S cm^−1^ at 150 °C. With 1.47 × 10^−1^ S cm^−1^ at 120 °C and 1.83 × 10^−1^ S cm^−1^ at 140 °C, the stability of proton conductivity measurements demonstrated the mechanical and component stability. The results indicate that the LBL self-assembly technique could be a promising approach for fabricating multilayered membranes as membrane electrolytes in high-temperature proton exchange membrane fuel cells.

Graphene oxide (GO) nanosheets have great mechanical qualities, a large surface area, and are inexpensive, making their application appealing. Liu K et al. proposed GO nanosheets coupled with electrospun polyvinyl chloride (PVC) nanofibers to form a sandwich structure membrane, or (PNs/GO/PNs)es [112]. By grafting GO nanosheets with imidazolium-based ionic liquids, imidazolium-GO (ImGO) nanosheets were also formed. By forming intermolecular hydrogen bonds with molecules of phosphoric acid (PA), the GO and ImGO nanosheets functioned as proton conduction carriers. The outside PVC nanofibers mat served as a shield for the inner layer of GO nanosheets and as a productive proton conduction medium. Due to quick proton conduction and a compact shape, the resultant PA-doped membranes showed better proton conductivity and improved mechanical characteristics. The maximal proton conductivities of the (PNs/GO/PNs)es/PA and PVC/ImGO/PA membranes, respectively, were 9.26 × 10^−2^ S cm^−1^ and 2.63 × 10^−2^ S cm^−1^ at 150 °C. The residual values after 384 h of continuous testing at 120 °C were 9.02 and 3.41 × 10^−2^ S cm^−1^, respectively. Notably, the tensile stress of the (PNs/GO/PNs)es/PA membrane was 11.6 MPa as opposed to the (PNs/GO/PNs)es membrane’s 7.11 MPa. In high-temperature proton exchange membranes (HTPEMs), this study shows that GO and ImGO nanosheets can improve proton conduction by lowering proton conduction resistance.

Huang L and his group used layer-by-layer self-assembly to build a multilayer structure of graphene oxide (GO) and sulfonated polyethersulfone (SPES) on polyester fiber mats by using the hydrogen-bonding interaction between GO and SPES [113]. Sulfonic acid groups were positioned in each layer along the fiber axis to provide long-range proton transmission channels, which aided in quick proton conduction. Their results show that with each successive assembly layer, the composite membranes’ proton conductivity increased. The composite membranes also have dramatically improved mechanical characteristics and methanol resistance. 

In addition to the above synthesis methods, there are a few other techniques, such as hot pressing and extrusion methods [114]. Cerium sulfophenyl phosphate (CeSPP), a newly developed proton conductor, was synthesized by combining m-sulfophenyl phosphonic acid (msPPA) and ammonium ceric nitrate. To create composite membranes, the CeSPP samples are blended with poly (2,5-benzimidazole) (ABPBI) using the polyphosphoric acid (PPA) hot-pressed method [115]. Notably, the proton conductivity of the membrane, doped with 38 wt% CeSPP, achieves a moderate value of 0.14 S cm^−1^ at 180 °C under 100% relative humidity. In a nutshell, the advantages and disadvantages of the preparation of nanomaterials are explained in Table 1. In addition, Table 2 gives a clear picture of various membranes and their characteristics. 

## 4. Properties of Nanomaterial Modified PEMs

### 4.1. Electric Properties 

For direct methanol FCs, a new hybrid PEM made of polyvinyl alcohol (PVA) and quaternized polyethyleneimine (QPEI) has been suggested by Zhang Y and his group [117]. QPEI is utilized as an anchoring agent to increase the compatibility of the organic-inorganic interface and decrease the leakage of phosphotungstic acid (PWA), which serves as the proton conductor. Due to hydrogen bonding and electrostatic interactions, the high solubility of PWA in water is decreased by using polymers as a substrate. After immersing in deionized water at 80 °C for 30 days, the optimized PVA-QPEI-2 PEM with PWA as consecutive proton transport channels achieved the highest proton conductivity achieved, which was 194.5 mS cm^−1^, and the residual proton conductivity was above 80%. At 60 °C and 40% relative humidity, the fuel cell built with the PVA-QPEI-2 PEM has a peak power density of 147.2 mW cm^−2^. Using a methanol permeability an order of magnitude lower than that of Nafion^®^ and easy fabrication, the organic-inorganic hybrid PEM using PWA as a proton carrier is extremely competitive for usage in DMFCs.

**Figure 12:**
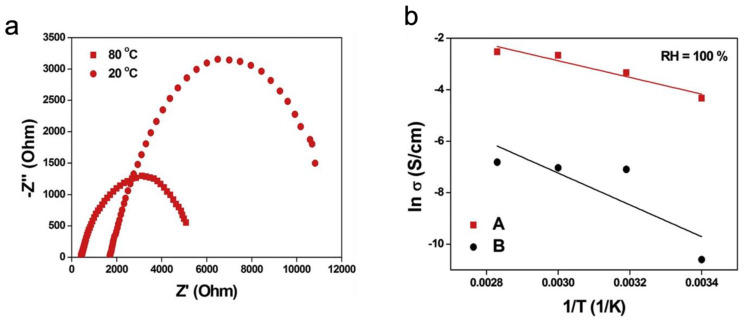
(**a**) The Nyquist plot of the impedance spectrum of F-TiO_2_-NT at 80 °C and 20 °C with 100% relative humidity. (**b**) Arrhenius plot for the conductivity of (**A**) F-TiO_2_-NT and (**B**) TiO_2_-NT at 100% relative humidity. Reprinted with permission from Ref. [118]. Copyright 2011 Elsevier.

In order to potentially improve the proton exchange membrane in high-temperature hydrogen polymer electrolyte membrane FC, functionalized titania nanotubes (F-TiO_2_-NT) were reported by Jun Y et al. [118]. As a sulfonic acid functionalization agent, 3-mercaptopropyl-tri-methoxysilane (MPTMS) was utilized to graft sulfonic acid groups onto the titania nanotubes (TiO_2_-NT). It was observed that F-TiO_2_-NT had a much better conductivity than TiO_2_-NT that had not been functionalized. Figure 12a shows the Nyquist plot of the impedance spectrum of F-TiO2-NT, and Figure 12b represents the Arrhenius plot for the conductivity of F-TiO_2_-NT and TiO_2_-NT. At 80 °C, F-TiO_2_-NT had a conductivity of 0.08 S cm^−1^, while non-functionalized TiO_2_-NT had a conductivity of about 0.0011 S cm^−1^. With a proton conductivity of 0.067 S cm^−1^ at 120 °C and 30% relative humidity at high temperature and low humidity, the composite membrane of F-TiO_2_-NT/Nafion exhibited robust proton conductivity.

When compared to a recast Nafion membrane, which only had a proton conductivity of 0.012 S cm^−1^ under the same circumstances, this was a significant improvement. F-TiO_2_-NT has a lot of promise as a membrane addition for high-temperature PEMFCs, according to these studies.

Feng K et al. devised a technique for evaporating graphene oxide sheets (GOSs) from their dispersion onto a distant aluminum foil surface to create rolled-up graphene oxide sheets [119]. The topological structure of the rolled-up GOSs on the aluminum foil surface can be altered by varying the amount of Al^3+^ ions that are produced as a result of the reaction among the alumina on the aluminum foil surface and the weak acidic condensed vapor of the GOS dispersion. Using this technique, the researchers created a GO/Nafion composite membrane for PEMFC, using the rolled-up GOSs as a support for the as-obtained hole-like self-assembled structure. In comparison to the recast Nafion membrane, the resulting composite membrane exhibits excellent proton conductivity, particularly in low-humidity situations. This is ascribed to the independent incorporation of rolled-up GOSs, which considerably enhance the proton transport by rearranging the microstructures of the Nafion matrix. 

Polybenzimidazole/Silicon dioxide (PBI/SiO_2_) hybrid membranes were developed in a work by Devrim, Y and co-workers and characterized in order to assess their potential as substitute materials for HT-PEMFC applications [120]. Laboratory-produced PBI polymer was used to create hybrid membranes that contained 5 wt% of SiO_2_ as an inorganic filler. SiO_2_ enhanced the proton conductivity and acid retention, with the PBI/SiO_2_ hybrid membrane achieving a maximum conductivity of 0.1027 S cm^−1^ at 180 °C. The anode and cathode of gas diffusion electrodes (GDE) were loaded with 1 mg Pt cm^−2^ catalyst using an ultrasonic coating process. The membranes were subjected to a single HT-PEMFC test employing a 5 cm^2^ active area running from 140 °C to 180 °C. The maximum current density was obtained for the PBI/SiO_2_ membrane at 165 °C with a cell voltage of 0.6 V, resulting in a current density of 0.24 A cm^−2^. The PBI/SiO_2_ hybrid membrane was shown to be more stable and performed better than the pure PBI membrane. These findings imply that PBI/SiO_2_ hybrid membranes have the potential to be improved fuel cell performance electrolytes for HT-PEMFC.

### 4.2. Thermal Properties 

Devrim Y et al. investigated the potential of PEMFCs using sulfonated polysulfone (sPS)/titanium dioxide (TiO_2_) composite membranes. Trimethylsilyl chlorosulfonate was used to sulfonate polysulfone (PS) at room temperature [121]. Levels of Sulfonation ranging from 15 to 40% were produced by adjusting the quantity of the sulfonating agent. The degree of sulfonation was raised with the molar ratio of the sulfonating agent to the PS repeat unit. Using the solution casting technique, composite membranes were created by combining TiO_2_ and sPS solution in DMAC (5 wt%). TiO_2_ was added, which enhanced the thermal stability; however, excessive filler concentrations declined the composite component’s miscibility and produced brittle membranes. The conductivity of the composite membranes ranged from 10^−3^ to 10^−2^ S cm^−1^, and it rose with operating temperature. The H_2_–O_2_/PEMFC running at 1 atm and 85 °C had the greatest characteristics of 300 mA cm^−2,^ with the sPS/TiO_2_ membrane at 0.6 V. sPS/TiO_2_ proved to be potential membrane material for PEM fuel cells, according to the results.

**Figure 13:**
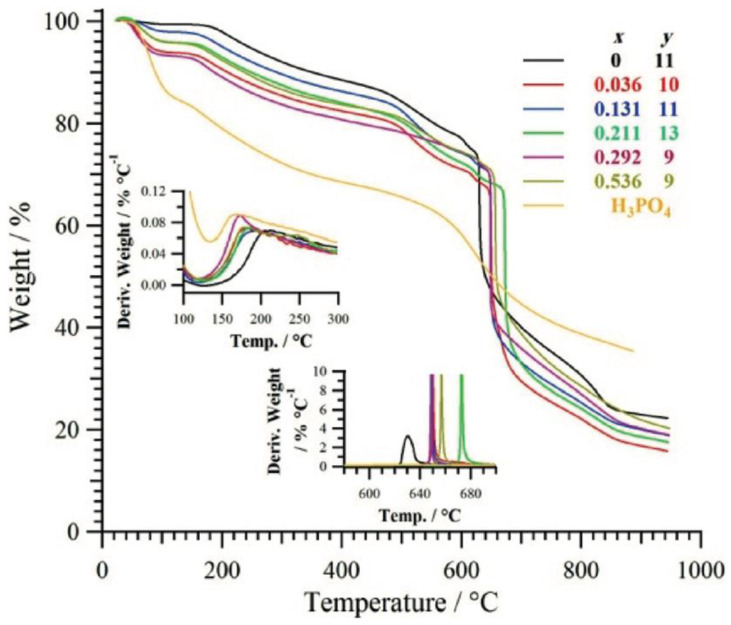
TG profiles for PBI4N(H_3_PO_4_)y, [PBI4N(ZrO_2_)x](H_3_PO_4_)y, and H_3_PO_4_(aq) measured under N_2_ from 30 to 9508C with the derivative d(wt%)/dT for two temperature ranges shown in the insets. Reprinted with permission from Ref. [122]. Copyright 2015 John Wiley and Sons.

Nawn G et al. designed PEM for fuel cells that can function at high temperatures (>100 °C) and exhibit conductivity in anhydrous environments [122]. To carry this out, poly[2,2′-(m-phenylene)-5,5′-bibenzimidazole] (PBI4N) composite membranes were prepared and investigated using a variety of methods. Results indicate that the characteristics of the membrane are significantly influenced by the amount of nanofiller present. While increasing it from 8 to 22 wt% has the opposite effect, increasing it from 0 to 8 wt% improves the acid uptake, thermal, and mechanical properties. TG profiles for PBI4N(H_3_PO_4_)y, [PBI4N(ZrO_2_)x](H_3_PO_4_)y, and H_3_PO_4_(aq) are shown in Figure 13. PBI4N(ZrO_2_)0.23113 has an ionic conductivity of 1.04 × 10^−1^ S cm^−1^, which suggests that the membrane has potential for usage in high-temperature proton exchange membrane fuel cells.

Shao ZG et al. looked at how well Nafion/silicon oxide and Nafion/silicon oxide composite membranes performed in proton exchange membrane fuel cells (PEMFCs) operating over 100 °C [123]. To prepare the composite membranes, Nafion solution was mixed with SiO_2_ and PWA/SiO_2_ mixtures. The findings showed that SiO_2_ and PWA were compatible with the Nafion membrane, and that their insertion into the membrane enhanced its initial degradation temperature and crystallinity. Additionally, compared to the Nafion recast membrane, the composite membrane had a higher water uptake. At high temperatures and 100% relative humidity (RH), the composite membranes’ proton conductivity was comparable to that of the original Nafion membrane, but it was much greater at low RH. The composite membranes, Nafion/SiO_2_/PWA and Nafion/SiO_2_, when used as an electrolyte in H_2_/O_2_ PEMFC, demonstrated higher current density values than the Nafion 115 membrane (95 mA cm^−2^) at an operating temperature of 110 °C and a humidified temperature of 100 °C (540 and 320 mA cm^−2^ at 0.4 V, respectively). This work implies that high-temperature PEMFC applications may be suitable for Nafion/silicon oxide composite membranes.

### 4.3. Structural Properties

In the research work by Maiti TK et al., the effects of combining SPEEK and sulfonated poly (benzimidazole) (SPBI) with propylsulfonic acid-functionalized graphene oxide (PrSGO) are examined [124]. By simulating several composite systems using atomistic molecular dynamics (MD), the effect of PrSGO loading on these characteristics was investigated. Tg was raised by cross-linking the polymer nanosystems and upping the percentage of PrSGO and SPBI loading in the polymer nanocomposite. Due to the robust interfacial connection between the PrSGO and the XSPEEK/SPBI matrix, the resulting XSPEEK/SPBI/PrSGO nanocomposite membranes displayed increased mechanical characteristics, chemical and thermal durability, and proton conductivity. The performance of the FC was enhanced overall as a result of the inclusion of PrSGO nanofillers in the polymer matrix. Proton conductivity significantly improved in a nanocomposite membrane with 4 wt% PrSGO loading, reaching 0.17 S cm^−1^ with 100% RH, 90 °C, and exhibited good FC performance with a maximum power density of 0.82 W cm^−2^ at 100% RH, 80 °C. This was ascribed to the acid group attached fillers’ superior interaction with the cross-linked SPEEK/SPBI-based matrix as well as their hygroscopic nature and increased amount of sulfonic acid groups.

Halloysite nanotubes (HNT), polyethersulfone (PES), and polyvinylpyrrolidone (PVP) composite membranes were produced using the electrospinning technique for use as proton exchange membranes in fuel cells by Eskitoros-Togay ŞM et al. [125]. Sulfonated HNT was used to adorn the PVP/sulfonated PES matrix, which led to the creation of PEMs. Proton transfer was improved, and proton conductivity values rose with temperature as the mass percentage of sulfonated HNT increased, as did swelling thickness, surface area, and water absorption capacity. Ion exchange capacity values rose up to 1% as the amount of sulfonated HNT increased. The sPES/PVP-sHNT-1.0 membrane had the greatest performance, with a greater current density of 450 mA cm^−2^ at 0.6 V than the competition. These nanofibrous composite membranes have a significant potential for usage in fuel cells, to sum up.

**Figure 14:**
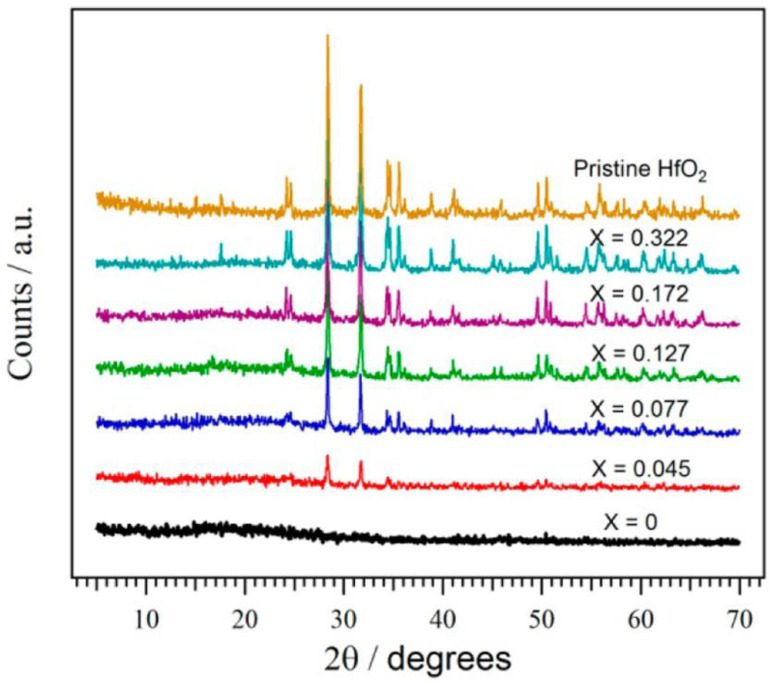
WAXS spectra of pristine PBI_4_N, [PBI_4_N(HfO_2_)x], and HfO_2_. Reprinted with permission from Ref. [126]. Copyright 2015 American Chemical Society.

PBI4N, also known as poly[2,2′-(m-phenylene)-5,5′-bibenzimidazole], has become a well-liked polymer for making high-temperature proton exchange membrane polymer fuel cells. Nawn G et al. describe the production of PBI4N composite membranes that have been doped with phosphoric acid and impregnated with hafnium oxide nanofiller at varying weight percentages (0–18 wt%) [126]. They examine the structure–property relationships of both the undoped and acid-doped composite membranes using thermogravimetric analysis, modulated differential scanning calorimetry, dynamic mechanical analysis, wide-angle X-ray scattering, infrared spectroscopy, and broadband electrical spectroscopy. Figure 14 clearly represents the structure determination using WAXS spectra of pristine PBI4N, [PBI4N(HfO_2_)x], and HfO_2_. According to their findings, adding nanofiller improves the thermal and mechanical characteristics of the undoped membranes and makes it easier for more acid to be taken in. PBI4N(HfO_2_)xy at 215 °C with x = 0.04 has a conductivity of 9.0 × 10^−2^ S cm^−1^ due to increased acid dissociation inside the acid-doped membranes caused by increasing the nanofiller concentration. The usage of these nanocomposite membranes in high-temperature PEMFCs has a lot of promise.

## 5. Future Scope of Nanomaterials Modified PEM

The future use of PEMs is bright and covers a variety of fields. Future developments that might occur include the following:

- Performance improvement: Improving PEM performance is one of the main goals. To increase efficiency and durability, scientists are creating novel materials, enhancing the electrolyte’s conductivity, and enhancing membrane structure.
- Cost reduction: The high price of fuel cells is one of the main obstacles to their broad implementation. Researchers are aiming to lower the cost of fuel cells by using inexpensive materials and new, less energy-intensive production techniques.
- Durability: The short lifespan of PEMs is another significant obstacle. New materials that are more resilient and can resist challenging operating circumstances are now being developed by researchers.
- Miniaturization: Another interesting development is the miniaturization of fuel cells. In order to power portable electronics, such as laptops, cell phones, and other electronic devices, researchers are striving to create miniature fuel cells.
- Integration: Another area of study is the integration of fuel cells with other energy storage technologies. For the purpose of developing hybrid energy storage systems that can serve as a dependable and sustainable source of power, researchers are looking into the feasibility of combining fuel cells with batteries and supercapacitors.

Future research objectives in PEMs modified with nanomaterials can essentially be in the area of synthesis of novel nanomaterials. There is still a need for the creation of novel, advanced nanomaterials that can provide superior qualities to those already in use. For PEM modification, researchers can investigate the usage of novel materials, including transition metal dichalcogenides (TMDs), metal-organic frameworks (MOFs), covalent organic frameworks (COFs), and 2D materials such as graphene. The development of novel manufacturing methods for PEMs enhanced with nanomaterials is yet another potential field of study. This may entail investigating novel means for adding nanoparticles to PEMs, such as electrospinning or layer-by-layer assembly approaches. Researchers might also look at the possible usage of PEMs modified with nanomaterials in applications other than fuel cells, such as water treatment or energy storage. This might entail researching the characteristics of various nanoparticle kinds and how they might be modified for certain uses.

Obtaining a better knowledge of the processes behind the behavior of PEMs modified with nanomaterials is another possible research objective. This might entail exploring how various kinds of nanoparticles interact with the membrane and simulating PEM behavior at the molecular level using sophisticated computer modeling tools. The possible consequences of PEMs modified with nanomaterials on the environment and human health might also be investigated by researchers, who could then devise mitigation plans. This might entail researching the possibility of nanoparticles contaminating the environment or endangering human health, as well as creating strategies for the secure disposal of these substances.

## 6. Summary

In summary, nanoparticles, such as carbon nanotubes, graphene oxide, titanium oxide, cerium oxide, zirconium oxide, polymer nanomaterials, and silica, have all been studied for usage in PEMs for fuel cells. These nanoparticles each have distinct qualities that may improve the performance characteristics of PEM, such as improved proton conductivity, increased mechanical strength, and increased resistance to deterioration. However, more investigation is required to fully comprehend how various nanoparticles affect PEM performance and to choose the best nanoparticle for a particular fuel cell application. PEMs are frequently synthesized by in situ polymerization, electrospinning, and solution casting. Each of these approaches has benefits and drawbacks in terms of price, scalability, and manufacturing simplicity. For instance, solution casting is a straightforward and inexpensive technique, but it might not be as scalable as other techniques and produce PEMs of inferior quality. Greater control over the polymerization process is possible with in situ polymerization, but it might cost more in terms of materials and equipment. Due to the need for specialized equipment, electrospinning can produce PEMs that are highly uniform and high in performance, but it may be more expensive. The most cost-effective synthesis technique for a particular PEM application may ultimately depend on a number of variables and may necessitate a trade-off between price, performance, and scalability.

## Figures and Tables

**Table 1:** Advantages and disadvantages of various synthesis methods of nanomaterials.

Synthesis Method	Advantages	Disadvantages
Sol-Gel Method	Versatile and enables fine composition and structure modification	Plenty of stages with complicated procedure
	Can include a variety of nanomaterials	Gel formation involves high temperatures
	Improves thermal and mechanical stability	Risk for nanoparticle agglomeration
Electrospinning	Produce fibers with a high surface area to volume ratio that are nanoscale	Minimal capacity to regulate fiber orientation and alignment
	Enables the inclusion of various nanoparticles	Mass production is only partially scalable.
	Has excellent mechanical characteristics	Vulnerable to fiber damage when handled
In situ Polymerization	Nanomaterials are dispersed uniformly	The ability to modify the size and shape of nanoparticles is inadequate
	Strong interfacial bonding between polymer matrix and nanomaterials	May require optimization of reaction conditions
	Enhances mechanical and thermal properties	Difficulty in achieving high nanoparticle loading
	Scalable and suitable for mass production	
Solution Casting	Quick and easy process	Inadequate control over the dispersion of nanoparticles
	For production on a large scale, scaling is simple	Possibility of nanoparticle loss during washing processes
	Economically feasible compared to other approaches	It could be necessary to perform post-treatment to incorporate all nanoparticles
Layer-by-Layer Assembly	Allows for precise oversight of the deposition of nanoparticles	A labor-intensive and tedious process
	Provides flexibility in layer composition and thickness	Large-scale production is only partially scalable
	Enables regulated and sequential deposition	Interfacial defect possibilities between layers
	Permits the development of many layers for superior properties	

**Table 2:** Comparison of various membranes and their properties.

Membrane	Preparation Method	Conductivity/Current Density	Temperature	Peak Power Density	Fuel	Ref.
PBI/SNP-PBI nanocomposites	Solution-casting	50 mS cm^−1^	160 °C	650 mW cm^−2^	Hydrogen	[60]
SPEEK/PVdF-HFP/SiO_2_	Solution-casting	8 × 10^−2^ S cm^−1^	90 °C	1.5 mW m^−2^	Microbial	[63]
SPEEK 6% W-TNT	Solution-casting	690 mA cm^−2^	80 °C	352 mW cm^−2^	Hydrogen	[66]
PA-doped PBI-sTP2	Spin coating	0.096 S cm^−1^	150 °C	621 mW cm^−2^	Hydrogen	[67]
Aquivion/TiO_2_/ZrO_2_	Impregnation	0.027 S cm^−1^	75 °C	1120 mW cm^−2^	Hydrogen	[69]
The Nafion-ZrNT	Solution-casting	140 mS cm^−1^	80 °C	982 mW cm^−2^	Hydrogen	[72]
CTS/BPO4@MWNT	Solution-casting	0.040 S cm^−1^	80 °C	49.0 mW cm^−2^	Methanol	[73]
SPEEK/ZSC	Solution-casting	38.10 mS cm^−1^	80 °C	38.9 mW cm^−2^	Methanol	[74]
CS/SSiO_2_@CNTs	Solution casting	35.8 mS cm^−1^	70 °C	60.7 mW cm^−2^	Methanol	[75]
NCC/PVA-SHGO-1.0	Solution casting	1.1 × 10^−2^ S cm^−1^	80 °C	31.4 mW cm^−2^	Hydrogen	[78]
PEM doped with 8% PS-MGO	Grafting	0.084 S cm^−1^	25 °C	78 mW cm^−2^	Methanol	[80]
polybenzimidazole (PBI)/sulfonated graphene oxide (sGO)	Solution casting	0.118 S cm^−1^	160 °C	364 mW cm^−2^	Hydrogen	[82]
PAP100-POSSI5.0	Solution casting	0.105 S cm^−1^	80 °C	152.37 mW cm^−2^	Hydrogen	[116]
Pi-POSS15%/Pi-SEBS	Solution casting	69.11 mS cm^−1^	80 °C	219 mW cm^−2^	Hydrogen	[92]
sPBT-E62.5/SGO3	In situ polymerization	0.139 S cm^−1^	80 °C	519.9 mW cm^−2^	Hydrogen	[93]
C-SPEEK/HPW/GO	In situ polymerization	119.04 mS cm^−1^	80 °C	876.80 mW cm^−2^	Hydrogen	[95]
CA-PTFE RCMs	Solution casting	0.210 S cm^−1^	80 °C	0.85 W cm^−2^	Hydrogen	[104]
SPEEK/cloisite fibre mats	Solution casting	7.73 mA cm^−2^	60 °C	1.18 mW cm^−2^	Methanol	[106]
ss-DNA@GO	Electrostatic layer-by-layer assembly	351.8 mS cm^−1^	80 °C	255.33 mW cm^−2^	Methanol	[109]
PU/CNT-CdTe/PU/CS)150/60%PA	Layer-by-layer assembly	6.82 × 10^−2^ S cm^−1^	150 °C	-	Methanol	[110]
PU/GO/PDDA/GO)200/60%PA	Layer-by-layer assembly	1.83 × 10^−1^ S cm^−1^	150 °C	-	Methanol	[111]
PNs/GO/PNs)es/PA	Layer-by-layer assembly	9.26 × 10^−2^ S cm^−1^	150 °C	-	Methanol	[112]

## Data Availability

Not applicable.
